# Ensemble learning model for identifying the hallmark genes of NFκB/TNF signaling pathway in cancers

**DOI:** 10.1186/s12967-023-04355-5

**Published:** 2023-07-20

**Authors:** Yin-Yuan Su, Yu-Ling Liu, Hsuan-Cheng Huang, Chen-Ching Lin

**Affiliations:** 1 Institute of Biomedical Informatics, National Yang Ming Chiao Tung University, Taipei, Taiwan; 2 Division of General Surgery, Department of Surgery, Kaohsiung Chang Gung Memorial Hospital, Kaohsiung, Taiwan

**Keywords:** Ensemble learning model, Carcinogenesis, Nuclear factor kappa B (NFκB), Tumor necrosis factor (TNF), Triple-negative breast cancer (TNBC), Precision medicine, Network medicine

## Abstract

**Background:**

The nuclear factor kappa B (NFκB) regulatory pathways downstream of tumor necrosis factor (TNF) play a critical role in carcinogenesis. However, the widespread influence of NFκB in cells can result in off-target effects, making it a challenging therapeutic target. Ensemble learning is a machine learning technique where multiple models are combined to improve the performance and robustness of the prediction. Accordingly, an ensemble learning model could uncover more precise targets within the NFκB/TNF signaling pathway for cancer therapy.

**Methods:**

In this study, we trained an ensemble learning model on the transcriptome profiles from 16 cancer types in the TCGA database to identify a robust set of genes that are consistently associated with the NFκB/TNF pathway in cancer. Our model uses cancer patients as features to predict the genes involved in the NFκB/TNF signaling pathway and can be adapted to predict the genes for different cancer types by switching the cancer type of patients. We also performed functional analysis, survival analysis, and a case study of triple-negative breast cancer to demonstrate our model's potential in translational cancer medicine.

**Results:**

Our model accurately identified genes regulated by NFκB in response to TNF in cancer patients. The downstream analysis showed that the identified genes are typically involved in the canonical NFκB-regulated pathways, particularly in adaptive immunity, anti-apoptosis, and cellular response to cytokine stimuli. These genes were found to have oncogenic properties and detrimental effects on patient survival. Our model also could distinguish patients with a specific cancer subtype, triple-negative breast cancer (TNBC), which is known to be influenced by NFκB-regulated pathways downstream of TNF. Furthermore, a functional module known as mononuclear cell differentiation was identified that accurately predicts TNBC patients and poor short-term survival in non-TNBC patients, providing a potential avenue for developing precision medicine for cancer subtypes.

**Conclusions:**

In conclusion, our approach enables the discovery of genes in NFκB-regulated pathways in response to TNF and their relevance to carcinogenesis. We successfully categorized these genes into functional groups, providing valuable insights for discovering more precise and targeted cancer therapeutics.

**Supplementary Information:**

The online version contains supplementary material available at 10.1186/s12967-023-04355-5.

## Background

Tumor necrosis factor (TNF) is a cytokine that regulates abundant critical cell functions, including cell proliferation, inflammation, differentiation, migration, and apoptosis [1, 2]. TNF has been implicated in various diseases [3] and cancers [2, 4]. Among the downstream factors of TNF, nuclear factor kappa B (NFκB) is a transcription factor that resides in the cytoplasm of cells and translocates into the nucleus to turn on gene transcription. TNF regulates this activation and further triggers the NFκB canonical pathway essential for cell proliferation, migration, inflammation, and anti-apoptosis [5, 6]. NFκB is ubiquitous and plays a crucial role in immune response and cancer formation. However, the aberrant activation of NFκB is frequently observed in various diseases [7–10] and cancers [11–13]. Due to its pivotal role as a "prime regulator" in inflammatory pathways, NFκB has been proposed as a therapeutic target for cancer by inhibiting NFκB to prevent the proliferation of cancer cells [14, 15]. However, targeting NFκB poses challenges as its inhibition affects cancer cell proliferation and suppresses immune responses in cancer cells and the survival of normal cells.

Additionally, NFκB exhibits sensitivity to external stimuli such as chemotherapy and radiation therapy, both of which inhibit apoptosis in cancer cells [15, 16]. Moreover, NFκB can be activated by diverse signals from receptors, including members of the TNF and IL-1 cytokine families, and engages in cross-talk with other transcription factors, signaling pathways, or miRNAs [17, 18]. These differently oriented impacts and sophisticated networks make developing NFκB-based drugs more challenging and require careful consideration to achieve effective and precise therapeutic interventions.

Numerous therapeutic strategies have been developed to inhibit NF-κB, a protein that can effectively treat malignancies [16]. However, many of these strategies are associated with adverse side effects, and some patients do not respond to the available therapies [18, 19]. Based on those circumstances, a more profound understanding of the mechanisms involved in the pathway of specific genes can help design effective therapeutic interventions. Various technological approaches have been proposed to advance progress in cancer therapy [20–22]. With advancements in computer hardware, biochemical technology, and the massive amount of biological datasets, scientists have made breakthroughs in this endeavor, and the application of machine learning has become an indispensable trend in various fields, including genetics, genomics [23], and proteomics [24]. In addition, machine learning techniques have been widely employed in cancer research. Supervised learning algorithms are the most prevalent in modern biomedical research for creating models that predict patients' risk factors, the presence of cancer, prognosis, and treatment outcomes. In essence, supervised learning algorithms analyze patterns or structures in input data, such as clinical or pathological images and molecular data, from cancer patients to build precise prediction models for diagnosis, early detection, and treatment decisions for future patients with uncertain cancer status. Several machine learning algorithms have received approval from the Food and Drug Administration (FDA) for practical clinical application. For example, Paige Prostate has been approved for the diagnosis of prostate cancer using biopsy slides [25], and Optellum has been approved for lung cancer detection via CT images [26].

Typical machine learning architectures rely on extensive data training to achieve reliable results. Accordingly, the generalizability of machine learning models developed for practical application is a significant concern, as there is often a lack of gold-standard tests or representative datasets for evaluation. Consequently, the inadequacy in data acquisition emerges as one of the most significant limitations in the development of machine learning techniques for medical applications and further makes the identification of potentially valuable biomarkers more challenging. In such cases, a semi-supervised learning approach can often be employed. A semi-supervised learning approach involves training the machine learning model using a well-known gene collection associated with specific diseases and then utilizing the model to identify other markers related or similar to the well-known gene set from unlabeled whole-genome data for further applications [23]. The one-sample support vector machine (SVM), Support Vector Data Description (SVDD), and autoencoder are semi-supervised learning approaches usually used to identify genes associated with the tested genes, which in this study are NFKB/TNF hallmark genes. However, these algorithms primarily focus on patterns within the positive samples, consequently requiring a large sample size. Conversely, the ensemble learning system capitalizes on the differences between positive and negative samples to discern patterns in positive samples, making it less reliant on a large positive sample size. Since hallmark gene sets in cellular systems are usually relatively small, typically numbering in the hundreds rather than thousands, the one-sample SVM, SVDD, and autoencoder may be less suitable compared to the ensemble learning model.

The NFκB/TNF signaling pathways play a crucial role in carcinogenesis and have widespread impacts on the living system. However, the abundant downstream effects exerted by these pathways often come with unexpected consequences. These hidden off-target effects make them unsuitable as direct therapeutic targets in cancer. To tackle this challenge, we propose an ensemble learning model that utilizes a semi-supervised learning algorithm to extract the putative genes regulated by NFκB in response to TNF. Specifically, our model takes the NFκB/TNF hallmark genes as positive samples and employs cancer patients as features to learn the transcriptomics pattern of NFκB/TNF hallmark across cancer patients. Accordingly, our model is designed to recognize the genes involved in the NFκB/TNF signaling pathways and carcinogenesis. Indeed, the downstream analyses show the model's accuracy in discovering genes within the NFκB/TNF signaling pathway and influential on carcinogenesis. Furthermore, we demonstrated the applicability of the proposed framework in cancer medicine in breast cancer as a case study. 

## Methods

A detailed description of mRNA expression profiles from 16 cancer types in TCGA, the hallmark and cancer-dysregulated (C6) genes from MSigDB [27], functional and survival analysis of the voted genes, enrichment analysis of gene sets and patient groups, classification of breast cancer subtypes, a prediction model for classifying patients' TNBC statuses, and the conventional approach to identify the genes of interest can be found in the Supplementary Materials and Methods section.

### Construction of the ensemble learning model

**Fig. 1:**
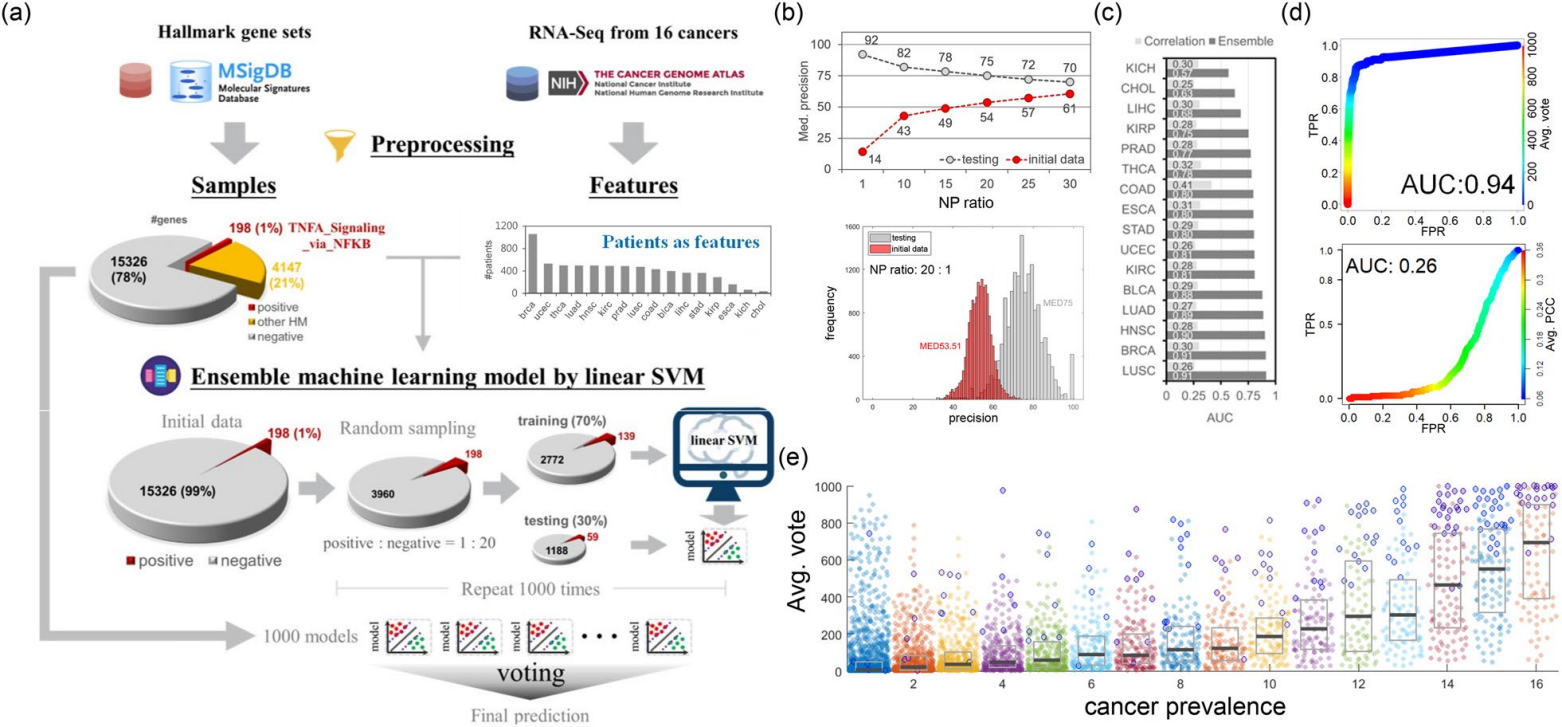
Performance of the ensemble learning model. **a** Overview of the model workflow. RNA-Seq data from patients diagnosed with 16 different types of cancers are used as features, while the NFκB/TNF hallmark gene sets are used as positive samples. The bar chart displays the number of patients per cancer type in descending order, and the pie chart represents the proportion of each gene subset. We trained 1000 member classifiers of linear SVM with an NP ratio of 20 to construct the final ensemble learning model for each cancer type. Finally, we applied the majority voting method that sums up the predictions from each member classifier to determine the tested genes' confidence. **b** Median precision for testing and initial data across all cancer types. The upper panel displays the median precision of the testing data (grey dashed line) and the initial data (red dashed line) at different NP ratios. At the NP ratio of 20, the median precision of the initial data surpasses 0.5, meeting the minimum requirements of a weak classifier. The lower histogram depicts the distribution of precision values from the initial (red) and testing (grey) data at the NP ratio of 20. **c** The area under the receiver operating characteristic curve (AUC) of the proposed ensemble model and the conventional correlation approach for each cancer type. The AUC values were calculated based on the false positive rate (FPR) and the true positive rate (TPR) obtained from the prediction of the 198 NFκB/TNF hallmark genes. **d** The receiver operating characteristic curve (ROC) for the pan-cancer ensemble model (upper panel) and conventional correlation approach (lower panel). **e** Distribution of the average votes of genes in each cancer prevalence. The average votes (Avg. vote) were calculated only from the cancer types that voted on the tested gene rather than all 16 cancers. The cancer prevalence is the number of cancer types in which the tested gene received votes. The median of average votes positively correlates with cancer prevalence. The data points with and without blue borders are the NFκB/TNF hallmark and non-NFκB/TNF hallmark genes, respectively. The genes with zero average votes were excluded here

This study aims to identify genes that are associated with the NFκB/TNF regulatory pathways and also have impacts on cancers. To achieve this goal, we utilized the NFκB/TNF hallmark gene set from MSigDB [27], which covers genes regulated by NFκB in response to TNF, as our positive data. The NFκB/TNF hallmark gene set contains 200 genes, and the RNA-Seq data from TCGA covered 198. These 198 NFκB/TNF hallmark genes were then used as positive samples during model training. MSigDB contains 50 hallmark gene sets, including the NFκB/TNF gene set, each representing certain biological states or processes. We excluded the 4147 genes belonging to the other 49 hallmark gene sets during the training process to avoid potential disturbances for model learning. Consequently, we considered the remaining 15,326 genes, which are not included in any hallmark gene sets, as the negative sample set. After model training, we applied the trained model to the complete transcriptome data, comprising 19,671 genes that include the previously excluded 4147 ones, to identify potential cancer-influential genes involved in the NFκB/TNF pathway. In summary, for each cancer type, we trained one cancer-specific ensemble learning model, resulting in 16 ensemble models. We then combined the prediction results of each gene from these 16 cancer-specific models to create a pan-cancer ensemble learning model. The workflow of the ensemble learning model is depicted in Fig. 1a, and the details for constructing our ensemble learning model are described as follows.

In our proposed model, the genes were considered as samples, while the patients from 16 cancer types served as features. It is important to note that the dataset consisted of 198 positive samples (the NFκB/TNF hallmark gene) and 15,326 negative samples (non-hallmark genes), resulting in a highly imbalanced ratio of negative to positive data (NP ratio), that is 99:1. We then employed the ensemble learning model that combines multiple member classifiers, typically weak, to build a strong ensemble classifier and can address the imbalanced class problem by adjusting the NP ratio for member classifiers. Considering the interpretability and reproducibility of the ensemble model for advanced analyses, we selected the linear support vector machine (SVM) algorithm as the basis for each member classifier. SVM utilizes support vectors, which are representative points, to construct a hyperplane for data classification. This choice of SVM allows for flexibility in discovering more candidate genes for effective integration and analysis. Other algorithms, such as neural networks, random forests, and logistic regression, were not selected due to their lower interpretability, reproducibility, or sensitivity to outliers in the data.

During the training process of the ensemble model, determining the sampling NP ratio is crucial. To address this issue, we employed a bagging strategy and randomly sampled data with various NP ratios: 1:1, 10:1, 15:1, 20:1, 25:1, and 30:1. For each cancer type, we trained 1,000 member classifiers for each ratio. During the training phase of each member classifier, we implemented a hold-out method that utilized 70% of the sampling data for training and reserved the remaining 30% for testing (Fig. 1a). We utilized the precision of testing data to evaluate the performance of each member classifier in predicting the NFκB/TNF hallmark gene for unseen data. In addition, we applied each trained member classifier to the initial data, which consists of 198 NFκB/TNF hallmark genes (positive) and 15,326 non-hallmark genes (negative). We then calculated the precision of the initial data to assess the overall performance of each member classifier in predicting the NFκB/TNF hallmark genes. Consequently, median precision from all the member classifiers across 16 cancer types, derived from the test and initial data, was employed to determine an appropriate NP ratio for the final ensemble model (Fig. 1b).

As expected, the median precision of the testing data decreased as the NP ratio increased. In contrast, the median precision of the initial data increased, suggesting that including more negative data improved the overall representation of information. At an NP ratio of 20, the median precision of the initial data exceeded 0.5; and the median precision of the testing data remained 0.75, which is acceptable (Fig. 1b). In ensemble learning models, a weak classifier usually performs poorly but should still outperform random guessing. The ratio of 20 met this requirement, as it performed better than random guessing on the initial data while maintaining stability on the testing data. Therefore, we used an NP ratio of 20 (3960 negatives: 198 positives) to train the member classifiers in the ensemble model (Fig. 1a and b). Finally, we employed the majority voting method to determine the confidence of the tested genes (samples). This method aggregated multiple weak classifications by counting the overall votes, representing the predicted probability of involvement in the NFκB/TNF pathway and its association with patient cohorts for each gene. Specifically, we trained 1000 classifiers for each cancer type to assemble the ensemble learning model, resulting in 16 ensemble models. In other words, for each gene, the maximal vote is 1000 in each cancer type. Additionally, we averaged the votes of each gene from the 16 cancer types to create a pan-cancer ensemble model. It is important to note that the average vote in the pan-cancer ensemble model is computed only from the cancer types that voted for the tested gene rather than from all 16 cancers.

## Results

### Accurate identification of NFκB/TNF hallmark genes in cancers using the ensemble learning model

In this study, we designed an ensemble learning model to identify genes involved in NFκB-regulated pathways downstream of TNF and carcinogenesis by using mRNA expression profiles of tumor patients as features. For each cancer type, we trained 1,000 classifiers to build an ensemble model specific to that single cancer. A pan-cancer ensemble model was also created by combining votes from the ensemble models of all 16 cancer types. The ensemble learning model assigns confidence scores to samples by aggregating votes from individual classifiers, thus revealing potential positive samples/targets. Our proposed method demonstrated high accuracy in identifying NFκB/TNF hallmark genes in 13 out of 16 cancer types (AUC ≥ 0.75, Fig. 1c) and performed even better in the pan-cancer model (AUC = 0.94, Fig. 1d). Compared to the conventional approach that used the Pearson correlation coefficient to identify a gene set with a high correlation to the 198 NFκB/TNF hallmark genes, our ensemble learning models demonstrated superior performance (Fig. 1c and d). Notably, the median precision of each member classifier for the initial data is around 0.5 (Fig. 1b), indicating that the outstanding performance is not due to overfitting.

The pan-cancer model identified NFκB/TNF hallmark genes implicated in multiple cancer types, as evidenced by their high prevalence across different cancer types and their corresponding high average votes (Fig. 1e and Additional file 1: Fig. S1). The average vote in the pan-cancer ensemble model is computed only from the cancer types that voted for the tested gene rather than from all 16 cancers. Among the 23 NFκB/TNF hallmark genes voted in all 16 cancer types, *EGR1*, *JUNB*, and *ZNF36* exhibited an average vote of 1,000. Previous studies have suggested that these genes play critical roles in tumorigenesis across cancers [28–30]. Similarly, the average vote of non-NFκB/TNF hallmark genes increases with their cancer prevalence (Fig. 1e). The non-NFκB/TNF hallmark genes refer to those genes not included in the NFκB/TNF hallmark gene set. Among the non-NFκB/TNF hallmark genes that received votes across all 16 cancer types, four genes (*SRGN*, *CCN2*, *TNFRSF12A*, and *ZFP36L1*) with an average vote exceeding 900 have been reported as regulated by TNF and NFκB [31–40]. That strongly suggests that they could participate in NFκB/TNF pathways. Furthermore, the involvement of these four genes in carcinogenesis has been observed across various cancer types [41–51]. These findings suggest that the genes with the higher average vote and cancer prevalence could be more reliable for being involved in TNFA/NFκB pathway.

### Newly identified candidate genes involved in the NFκB-regulated pathways downstream of TNF in cancer progression

**Fig. 2:**
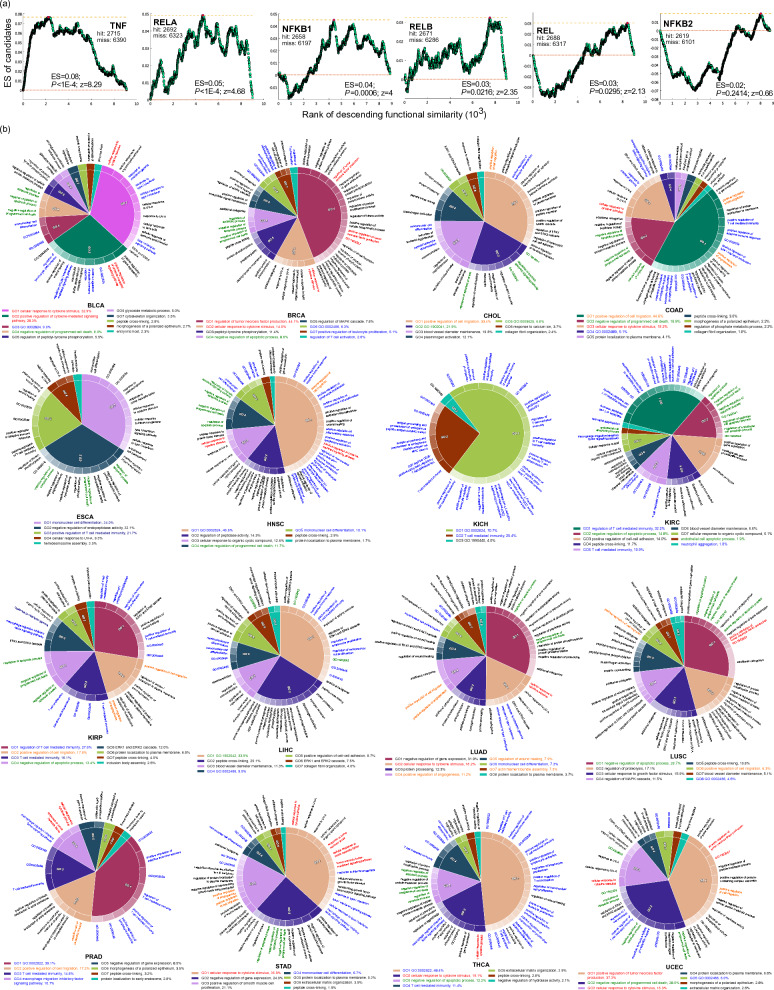
Functional analysis of the predicted candidates. **a** Functional similarity between the six core member genes and the predicted candidates. For each core member gene, only those genes having nonzero similarity to the corresponding core member gene were used in GSEA. We ranked these genes (x-axis) based on their functional similarity to the corresponding core member gene to calculate the enrichment score (ES, y-axis) by hitting the tested gene set-the predicted candidates. The number of hits in the candidate gene set is denoted as 'hit,' while 'miss' represents the number of genes without hits. The *p*-values (*P*) and z-scores were derived from 10,000 random permutations of gene ranking during the GSEA process. The maximal enrichment scores (ES) are marked as red circles. **b** Highly-voted functional modules within the candidates for each cancer type. The details of identifying the highly-voted functional modules formed by the predicted candidates in each cancer type are described in the Supplementary Information. Each pie chart shows the functional modules in which the highly voted candidates are involved in the corresponding cancer type. The percentage denotes the relative significance of one functional module to the others. Color coding is used to highlight functional modules associated with notable biological processes: red (positive regulation of tumor necrosis factor production or cellular response to cytokine stimulus), blue (immune response), green (negative regulation of cell death), and orange (cancer metastasis). The biological processes with long descriptions are represented by their GO terms (Additional file 1: Table S2)

Upon validating the accuracy of our ensemble model in identifying the NFκB/TNF hallmark genes, we further investigated the involvement of the newly identified candidate genes in the NFκB-regulated pathways downstream of TNF. In this context, the newly identified candidate genes refer to those non-NFκB/TNF hallmark genes that received at least one vote across all cancer types, hereafter referred to as ‘candidates’. Additional file 1: Table S1 records the precise number of genes with/without votes for each cancer type. We evaluated the functional similarity between the candidates and six central genes of the TNF and NFκB family (TNF, NFKB1, NFKB2, REL, RELA, and RELB) [52]. Specifically, we applied the Jaccard index, calculated based on the proportion of the shared experimentally validated biological processes between two genes, to assess the functional similarity between each gene in the RNA-Seq data and the six central genes of the TNF and NFκB family. The candidates showed significant enrichment in the high functional similarity region of *TNF* and *RELA* (Fig. 2a). Additionally, the candidates showed substantial enrichment scores for functional similarity to *NFKB1*, *RELB*, and *REL*, although they situated in the middle functional similarity region (Fig. 2a). However, the candidate genes showed lower functional similarity to *NFKB2* and exhibited insignificant enrichment (Fig. 2a). When comparing the candidates to the non-candidate genes (those non-NFκB/TNF hallmark genes receiving no votes across all cancer types), the candidates displayed higher similarity to the canonical pathway genes *TNF*, *RELA*, *NFKB1*, and *RELB* (Table 1). However, there is almost no difference in similarity to the *REL* gene and significantly lower similarity to the non-canonical pathway gene *NFKB2* (Table 1). These findings align with the results observed from the enrichment analysis (Fig. 2a). In contrast, the conventional correlation-based approach did not identify genes with significantly higher functional similarity to any of the core member genes. (Additional file 1: Fig. S2). These results highlight the effectiveness of our ensemble model in identifying genes involved in the canonical NFκB-regulated pathways, which are known to be downstream of TNF [5, 6, 14].

**Table 1:** Comparison of functional similarity

BP similarity	Candidate (#)	non-candidate(#)	*p*-value	Cohen’s *d*
TNF	0.0685 (2715)	0.0558 (6390)	6.79E-15	0.24
RELA	0.1092 (2692)	0.0994 (6323)	3.21E-07	0.11
NFKB1	0.1056 (2658)	0.1023 (6197)	2.57E-03	0.04
RELB	0.0851 (2671)	0.0831 (6286)	3.11E-02	0.04
REL	0.1599 (2688)	0.1706 (6317)	9.25E-01	− 0.07
NFKB2	0.1680 (2619)	0.1999 (6101)	1.08E-04	− 0.15

* BP* Biological Processes

(#) represents the number of genes with non-zero similarity, excluding NFκB/TNF hallmark genes

The similarity is evaluated using the Jaccard index, which measures the shared experimentally validated biological processes with the central member genes. The table presents each category's average BP similarity of the (#) genes

The *p*-values are derived from the Wilcoxon rank-sum test

To demonstrate the involvement of the predicted candidates in the canonical NFκB-regulated pathways, we performed a network-based functional enrichment analysis combined with gene set enrichment analysis (GSEA). This analysis aimed to identify functional modules formed by the highly-voted candidates in each cancer type, shedding light on their potential biological functions. To note, we performed GSEA by ranking the candidates based on their votes in the corresponding cancer type to hit the genes in the tested functional module to calculate the enrichment score. Our results showed that the functional modules involved in the positive regulation of tumor necrosis factor production or cellular response to cytokine stimulus were present in ten of the sixteen cancer types (Fig. 2b, red labels). This observation strengthens the hypothesis that the highly voted candidates play a significant role in the NFκB-regulated pathways downstream of TNF. Besides, across the sixteen cancer types, the most prevalent functions were related to the immune response (Fig. 2b, blue labels). Previous studies have indicated that the canonical NFκB-regulated pathways predominantly regulate innate and adaptive immunity [53]. Notably, the highly voted candidates were associated with adaptive immunity, such as T-cell mediated immunity and antigen processing and presentation of endogenous peptide antigen via MHC class I via ER pathway. These functions rely on the canonical NFκB pathway rather than the non-canonical one [54, 55].

In addition, functional modules involved in anti-apoptotic processes, such as negative regulation of apoptotic process or programmed cell death, were found in fourteen studied cancer types, except for KICH and PRAD (Fig. 2b, green labels). These findings are in harmony with previous studies indicating that the canonical NFκB pathway, when activated by TNF and other proinflammatory factors, promotes carcinogenesis by inhibiting apoptosis [12, 56, 57]. Furthermore, functional modules linked to cancer metastasis, including positive regulation of cell migration and positive regulation of angiogenesis, were found in nine studied cancer types (Fig. 2b, orange labels). That supports the idea that the canonical NFκB pathways downstream of TNF regulate angiogenic genes to trigger angiogenesis and promote metastasis [58]. Together, these results demonstrate the involvement of the highly voted candidates in the canonical NFκB-regulated pathway and highlight their potential oncogenic role in cancer progression.

### The ensemble model can identify cancer-dysregulated and poor-prognostic genes in cancers

**Fig. 3:**
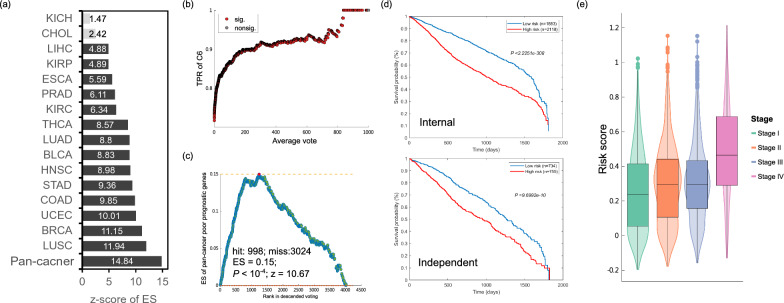
Oncogenicity and prognostic impact of the identified genes. **a** Enrichment analysis of oncogenic genes (C6) for each cancer and pan-cancer model's identified (voted) genes. Z-scores were calculated based on 10,000 random permutations generated in the GSEA process, wherein genes were ranked by votes to hit oncogenic genes in the C6 gene set. **b** Positive correlation between the proportion of oncogenic genes and average votes. The x-axis denotes the average votes of predicted genes across 16 cancer types. Each point represents the proportion of oncogenic genes with an average vote equal to or greater than the corresponding threshold on the x-axis. The red and gray circles indicate significant and insignificant enrichment, respectively, as determined by Fisher's exact test. **c** Enrichment analysis of pan-cancer poor-prognostic genes for identified genes of pan-cancer model. Herein, the identified genes were ranked by their average votes in the pan-cancer model to hit the pan-cancer poor-prognostic genes. Z-score and *p*-value were calculated from 10,000 random permutations of genes’ average vote. The number of hits in the poor-prognostic gene set is denoted as 'hit,' while 'miss' represents the number of the identified genes without hits. To note, among the 4678 identified genes in the pan-cancer model, only 4022 genes are provided with a pre-calculated hazard ratio (the exponential regression coefficient) in the Cox regression model from our previous study. **d** Kaplan–Meier plots (KM plot) of 5-year survival based on risk score calculated from the identified genes. The risk scores were calculated by combining the identified genes' expression levels with their coefficients in a pre-trained pan-cancer Cox-regression model. We classified patients into low and high-risk groups based on the median risk score. Kaplan–Meier plots were generated for both the internal (cancer types included in our ensemble learning model) and independent (cancer types not included in our ensemble learning model) datasets to evaluate the impact of the identified genes on patient survival. The number of patients in each group is indicated by "*n*" in the plots. **e** Risk score of patients in different stages. This figure illustrates the progression of patients’ risk scores across different pathologic stages. It exhibits a trend where the risk scores increase sequentially from stage I to stage IV: stage I < stage II < stage III <  < stage IV

We have illustrated that the ensemble model highlights candidate genes implicated in the canonical NFκB-regulated pathways downstream of TNF, and it uncovers their potential oncogenic roles through functional module analysis. Subsequently, we delved deeper into whether the genes identified by our ensemble model (voted genes) collectively contribute to carcinogenesis. The term "voted genes" is hereafter used to describe genes that received at least one vote across all cancer types, including those hallmark genes. Our findings show that cancer-dysregulated genes, obtained from the oncogenic gene set (C6) in MSigDB [27], are significantly enriched in the voted genes (71.72%, *p*-value = 5.90 × 10^–171^, Fisher's exact test). Cancer-dysregulated genes also earned more votes within individual cancer types, except for CHOL and KICH showing moderate significance, and had higher average votes from the pan-cancer model across cancers (Fig. 3a and Additional file 1: Fig. S3). That indicates that genes with higher votes are more likely to be dysregulated in cancer.

Furthermore, our results also show that the proportion of cancer-dysregulated genes increases with the vote threshold, demonstrating a positive correlation between votes and the potential oncogenicity of the identified genes (Fig. 3b and Additional file 1: Fig. S4). In contrast, the genes identified by the conventional correlation-based approach did not consistently exhibit significant oncogenicity across cancers or within each type (Additional file 1: Fig. S5 and S6). These findings confirm the oncogenicity of the voted genes and suggest that genes with higher votes have a greater likelihood of being involved in carcinogenesis.

Subsequently, to further understand the roles of voted genes in cancer progression, we explore their impact on patient survival using a pan-cancer survival influential gene set compiled by our previous study [59]. We observed that genes linked to poor survival outcomes are significantly overrepresented among the genes identified by the pan-cancer model (24.99%, *p*-value = 2.56 × 10^–43^, Fisher's exact test). These poor-prognostic genes also exhibit a significant enrichment among genes with more votes (Fig. 3c). It is noteworthy that genes with larger z-scores of estimated survival hazard ratio have a higher likelihood of being voted on by the pan-cancer ensemble model (*z* = 15.04, *p*-value = 3.97 × 10^–51^, logistic regression with Wald test), implying a connection between highly-voted genes and poor patient survival, indicative of their potential oncogenic nature. Notably, the poor-prognostic genes were significantly overrepresented among the 198 hallmark genes (41.92%, *p*-value = 8.11 × 10^–23^, Fisher's exact test), suggesting that the detrimental characteristics of the genes identified by the pan-cancer model may be inherited from the NFκB/TNF hallmarks. Moreover, our findings show that patients with higher risk scores calculated by these genes identified by the pan-cancer model exhibit poor prognoses in both internal and external datasets (Fig. 3d), underscoring the predictive prognostic potential of these genes. Furthermore, patients in stage IV possess significantly higher risk scores than those in other stages (Fig. 3e), indicating a potential role of these genes receiving votes from the pan-cancer model in cancer metastasis. Overall, these results strongly support the oncogenic potential of the voted (identified) genes in carcinogenesis and highlight their ability to predict the prognosis of cancer patients.

### An application of the ensemble model to identify subtypes and to predict the prognosis of breast cancer patients

**Fig. 4:**
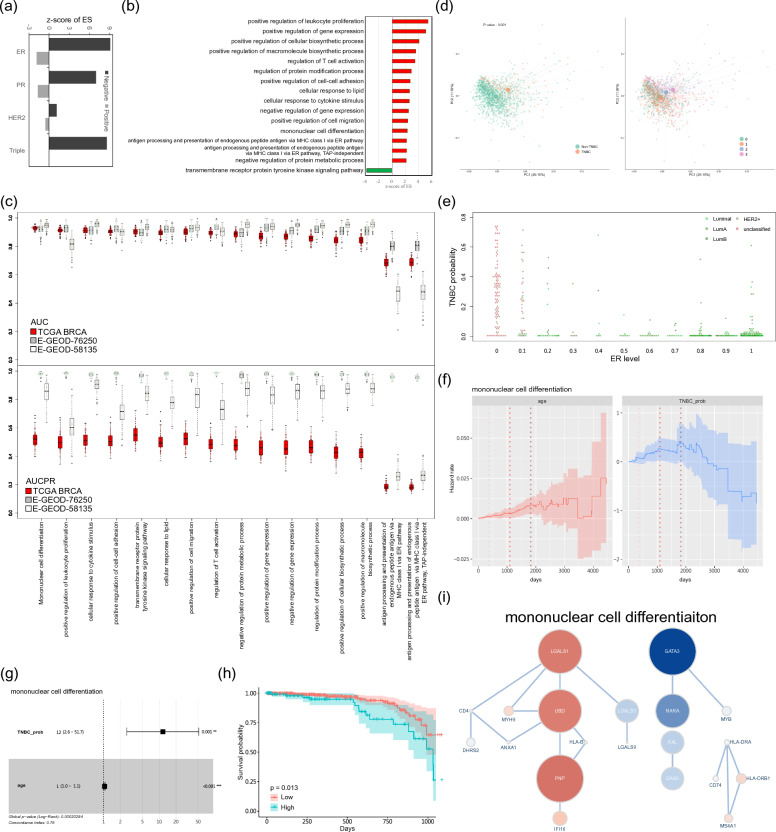
Triple-negative breast cancer analysis. **a** Enrichment analysis of breast cancer subtypes for patients by their weights in the BRCA ensemble model. The z-score of each subtype was calculated through 10,000 random permutations during the GSEA process, wherein patients were ranked by their weights in the BRCA model to hit patients with different subtypes. **b** The highly voted functional modules activated/inactivated in TNBC patients. The z-score, calculated from 1000 random permutations during the GSEA process, reflects the functional module's activity in TNBC patients. We assessed the activity of the highly-voted functional modules identified in BRCA by performing GSEA, wherein genes were ranked by their fold-change (TNBC vs. non-TNBC patients) to hit the member genes in the tested module. The modules marked in red indicate activated, while those marked in green indicate inactivated in TNBC patients. **c** The performance of identified functional modules in identifying TNBC patients. We performed 100 hold-out processes for each module to assess the potential overfitting. During the hold-out process, 60% of the sampling data was used for training, and the remaining 40% was used for testing. The performance metrics shown here are AUC and AUCPR values derived from the testing data. **d** Principal component analysis (PCA) of BRCA samples by expression profiles of the functional module “mononuclear cell differentiation”. The left panel illustrates the separation of TNBC and non-TNBC patients based on the expression profiles of the functional module, with the p-value obtained through a permutational multivariate analysis of variance (PERMANOVA). The right panel displays the trajectory of the receptor status in BRCA patients. **e** The association between the TNBC probability and non-TNBC patients’ ER level. The TNBC probabilities of all BRCA patients were predicted by a naïve logistic regression model using gene expression profiles in the mononuclear cell differentiation module. The subtypes of non-TNBC patients are displayed by colors. **f** Aalen’s additive regression model estimated the hazard rate of non-TNBC patient survival. The patients’ age and predicted TNBC probability are covariates in the model. The three vertical dash lines represent the time point of one, three, and five years from left to right. **g** The 3-year hazard ratio of age and predicted TNBC probability for non-TNBC patients. The hazard ratios were calculated by a multivariate Cox regression model using age and TNBC probability as covariates. **h** The Kaplan Meier survival curve of the estimated three-year survival probability of non-TNBC patients with high and low predicted TNBC probability. The *p*-value is estimated by the log-rank test. **i** The functional module of mononuclear cell differentiation. The size of each node in the figure represents the magnitude of the gene's impact on TNBC prediction. Red and blue nodes represent genes with positive and negative z-scores, respectively. Genes with an absolute z-score greater than two are labeled in white

To further show the model's applicability in cancer medicine, we selected triple-negative breast cancer (TNBC) as a case study. This selection was motivated by the fact that TNBC has been reported to be strongly associated with the dysregulation of NFκB/TNF [60–63]. Within the ensemble learning model, patients bearing larger absolute weights have more influence on predicting NFκB/TNF hallmark genes, indicating a potential connection to the NFκB/TNF pathway. Our results show that patients with larger weights in the model are significantly overrepresented in TNBC patients (Fig. 4a and Additional file 1: Fig. S7). Furthermore, these highly weighted patients are significantly overrepresented in ER-negative and PR-negative patients but significantly underrepresented in ER-positive and PR-positive patients (Fig. 4a and Additional file 1: Fig. S7). A similar, but not significant, correlation was also observed between the highly weighted patients and their HER2 status (Fig. 4a and Additional file 1: Fig. S7). These findings suggest that patients with larger weights in the model may be sensitive to ER and PR status and the NFκB/TNF pathway dysregulation. This conclusion is supported by previous studies indicating the mutual suppression between NFκB/TNF and ER/PR in breast cancer [60–63]. Moreover, the ER + /PR + patients receiving endocrine therapy could have a better prognosis by repressing NFκB [60–63]. This recapitulates the association between TNBC and the NFκB/TNF pathway. Briefly, these results support the reliability and interpretability of our model, suggesting that the model can recognize patients with subtypes of breast cancer that are highly associated with NFκB/TNF genes.

To further demonstrate the effectiveness of our model in exploring TNBC, we uncovered the functional modules that are significantly enriched with highly-voted candidates in the BRCA ensemble model and significantly activated or inactivated in TNBC patients (Fig. 4b and Additional file 1: Table S3). We determined the status of these modules in TNBC patients by evaluating the activity of the functional modules identified in BRCA (Fig. 2b, BRCA panel) through GSEA that used fold-change of gene expression level as gene rank. Each gene's fold change was calculated from the differential expression analysis between TNBC and non-TNBC implemented by the Limma-Voom R package [64]. Out of the sixteen identified functional modules, fifteen were up-regulated, and one was down-regulated in TNBC patients; one-third of the up-regulated modules are involved in the immune process. As anticipated, these functional modules can accurately predict TNBC patients in the TCGA dataset, excluding two MHC class I-related modules containing only three genes for each (Fig. 4c and Table 2). However, the area under the precision-recall curve (AUCPR) from the TCGA dataset was only satisfactory (Fig. 4c and Table 2). Notably, the identified functional modules can also accurately predict TNBC patients in two external datasets with higher AUCPR values than the TCGA dataset (Fig. 4c and Table 2). This robust performance highlights the potential of these modules as promising diagnostic network biomarkers for determining TNBC status in breast cancer patients.

**Table 2:** Performance of the identified modules in predicting TNBC

Module	#genes	TCGA		E-GEOD-76250		E-GEOD-58135	
		AUC	AUCPR	AUC	AUCPR	AUC	AUCPR
Mononuclear cell differentiation	20	0.93 ± 0.01	0.51 ± 0.05	0.93 ± 0.03	0.98 ± 0.01	0.95 ± 0.03	0.85 ± 0.08
Positive regulation of leukocyte proliferation	15	0.91 ± 0.02	0.50 ± 0.06	0.93 ± 0.03	0.99 ± 0.01	0.83 ± 0.05	0.63 ± 0.09
Cellular response to cytokine stimulus	52	0.91 ± 0.02	0.51 ± 0.05	0.91 ± 0.04	0.98 ± 0.02	0.95 ± 0.03	0.89 ± 0.07
Positive regulation of cell–cell adhesion	27	0.91 ± 0.02	0.52 ± 0.06	0.92 ± 0.04	0.98 ± 0.02	0.90 ± 0.03	0.71 ± 0.08
Cellular response to lipid	36	0.90 ± 0.02	0.55 ± 0.06	0.91 ± 0.04	0.97 ± 0.02	0.94 ± 0.03	0.85 ± 0.07
Transmembrane receptor protein tyrosine kinase signaling pathway	46	0.90 ± 0.03	0.51 ± 0.06	0.92 ± 0.04	0.98 ± 0.01	0.92 ± 0.03	0.78 ± 0.09
Positive regulation of cell migration	78	0.90 ± 0.02	0.52 ± 0.07	0.93 ± 0.04	0.98 ± 0.02	0.94 ± 0.03	0.79 ± 0.10
Regulation of T cell activation	21	0.90 ± 0.02	0.48 ± 0.06	0.94 ± 0.03	0.99 ± 0.01	0.91 ± 0.05	0.73 ± 0.10
Negative regulation of protein metabolic process	90	0.89 ± 0.03	0.48 ± 0.06	0.90 ± 0.03	0.97 ± 0.02	0.95 ± 0.02	0.87 ± 0.07
Positive regulation of gene expression	112	0.88 ± 0.03	0.45 ± 0.06	0.93 ± 0.03	0.98 ± 0.02	0.94 ± 0.03	0.83 ± 0.07
Negative regulation of gene expression	88	0.87 ± 0.03	0.45 ± 0.07	0.92 ± 0.03	0.98 ± 0.01	0.95 ± 0.02	0.86 ± 0.07
Regulation of protein modification process	117	0.85 ± 0.03	0.48 ± 0.05	0.94 ± 0.04	0.99 ± 0.02	0.95 ± 0.02	0.86 ± 0.07
Positive regulation of cellular biosynthetic process	147	0.84 ± 0.03	0.42 ± 0.06	0.91 ± 0.04	0.98 ± 0.02	0.95 ± 0.02	0.88 ± 0.06
Positive regulation of macromolecule biosynthetic process	139	0.84 ± 0.03	0.43 ± 0.05	0.91 ± 0.04	0.98 ± 0.02	0.95 ± 0.02	0.87 ± 0.05
Antigen processing and presentation of endogenous peptide antigen via MHC class I via ER pathway	3	0.69 ± 0.04	0.19 ± 0.02	0.81 ± 0.04	0.96 ± 0.01	0.48 ± 0.08	0.26 ± 0.05
Antigen processing and presentation of endogenous peptide antigen via MHC class I via ER pathway, TAP-independent	3	0.69 ± 0.03	0.18 ± 0.02	0.80 ± 0.05	0.96 ± 0.01	0.48 ± 0.08	0.25 ± 0.04

Furthermore, this outstanding performance suggests that non-TNBC patients in the TCGA dataset with a high probability of TNBC predicted by our model could exhibit similar transcriptome characteristics to TNBC patients. To further examine these non-TNBC patients, we investigated the best-performing functional module, mononuclear cell differentiation, for TNBC prediction. Gene expressions in this module effectively distinguished TNBC status and the trajectory of receptor status in patients (Fig. 4d). Non-TNBC patients with luminal breast cancer, who expressed estrogen receptors, were predicted with lower probabilities of being TNBC (Fig. 4e). On the other hand, patients with lower or zero estrogen receptor levels, such as those classified as HER2 enriched or unclassified by immunohistochemistry staining, tended to be predicted with higher probabilities of TNBC (Fig. 4e). This observation suggests that gene expressions in the module could be associated with the molecular subtyping of breast cancer.

Moreover, the predicted probability of these non-TNBC patients has a significant effect on the hazard rate of death in the first three years while having a decreasing effect after five years (Fig. 4f). Notably, while the hazard rate of age positively correlates with the survival duration, the magnitude of the hazard rate associated with age is less than that of the predicted TNBC probability. This observation indicates that the predicted TNBC probability has a more considerable impact on patient prognosis than age. The hazard ratio of predicted probability is 12 on 3-year overall survival (Fig. 4g), indicating that non-TNBC patients with higher predicted probability have an increased risk of dying within the first three years. Using a predicted probability cut-off of 0.05, which is top 20% of non-TNBC patients, the patients with higher probability have significantly lower 3-year overall survival rates (Fig. 4h). This result implies that non-TNBC patients with higher predicted probability may have progressive breast cancer and a higher risk of short term mortality. However, when using genes as covariates instead of predicted probability, only a few genes significantly affected the non-TNBC patient mortality hazard ratio (Additional file 1: Fig. S8). That suggests that these genes must work as a module, not individually, to affect patient survival.

Accordingly, targeting the pivotal node in the network has the potential to disrupt its molecular function and hinder tumorigenesis. LGALS1 and UBD are the top two hubs in the mononuclear cell differentiation module with the highest degree and most significant effect size in predicting TNBC (Fig. 4i). Previous study has indicated that UBD (FAT10/Ubiquitin D) promotes the invasion of the progressive breast cancer cell by stabilizing ZEB2 [65]. Galectin-1 (LGALS1) has been reported to be associated with the metastatic potential of breast cancer [66, 67]. Inhibition of LGALS1 has been shown to reduce the metastatic capability of the MDA-MB-231 breast cancer cell line [68], and expression of LGALS1 can affect the metastasis of breast cancer, supporting our hypothesis that targeting LGALS1 could obstruct tumor progression. In addition, UBD regulates the TNF-induced NFκB activation in immune response [69], and LGALS1 has been found to participate in TNF/ NFκB-regulated inflammation [70, 71]. Notably, these two genes were identified as candidate genes by our ensemble learning model but not recognized as the NFκB/TNF hallmark genes in the MsigDB, highlighting the ability of our approach to uncover promising genes in NFκB-regulated pathways downstream of TNF. In conclusion, the "mononuclear cell differentiation" functional module has significant potential for predicting the prognosis of breast cancer and hindering the mechanism of breast cancer metastasis.

## Discussion

In this study, we proposed an ensemble learning model that can accurately identify the genes involved in NFκB-regulated pathways downstream of TNF and carcinogenesis. Our ensemble learning method addresses the challenge of imbalanced data by utilizing bootstrap sampling, which helps decentralize samples with uncertain status and improve the predictions' robustness. We determined a proper sampling ratio through a systematic process, as depicted in Fig. 1b. Another critical consideration in our study is the number of member classifiers in the ensemble model. To ensure unbiased predictions and maintain model performance and rationality, we constructed 16 independent ensemble models for 16 cancer types, each comprising 500 classifiers. This analysis reveals a strong correlation (Spearman's *ρ* ranging from 0.97 to 0.99 across the 16 cancers) between gene votes in the original ensemble models and those in the independent models with 500 member classifiers (Additional file 1: Fig. S9). This finding underscores the stability and robustness of our model and validates the sufficiency of using 1,000 member classifiers to capture the overall information for the studied cancers. Our ensemble learning model’s exceptional stability and performance indicate that it can effectively identify credible genes involved in the NFκB/TNF pathway.

The analysis also revealed that the number of member classifiers is a potential limitation of the ensemble learning model. Having too few member classifiers can result in the model being unable to capture the comprehensive information within the samples. Conversely, too many member classifiers can lead to excessive computational demands, reducing the model's efficiency. Another factor that could limit the performance of the ensemble learning algorithm is the balance between features. For example, in constructing a pan-cancer ensemble learning model, simply combining all patients (features) from multiple cancers may not be ideal, as the uneven distribution of cancer types could confound the model or cause the results to be skewed towards cancer types with larger sample sizes. This is also a major reason why we chose the current strategy for constructing the pan-cancer model in this study.

On the other hand, the NFκB/TNF hallmark genes exhibit higher average votes and higher cancer prevalence across multiple cancer types (Fig. 1e), implying that the predicted candidates with more votes and greater cancer prevalence are more likely to be reliable in terms of their pan-cancer association with the NFκB/TNF pathway. Indeed, we highlighted three pan-cancer NFκB/TNF hallmark genes (*EGR1*, *JUNB*, and *ZNF36*) [28–30] and identified four candidates (*SRGN*, *CCN2*, *TNFRSF12A*, and *ZFP36L1*) that are highly potentially involved in NFκB/TNF pathways in various cancers [31–40]. Conversely, genes with high votes but low cancer prevalence may be involved in the NFκB/TNF pathway in a cancer-specific manner. For instance, *STAT5*, an NFκB/TNF hallmark gene, received 744 votes exclusively in BRCA. Previous studies have linked *STAT5A*, also known as mammary gland factor (MGF), to mammary function and its high expression in human breast cancers [72, 73]. These results suggest a substantial correlation between *STAT5A* and breast cancer, potentially suggesting its specificity to breast cancer.

Another two examples are *C9* and *G6PC*, which are two candidate genes that obtained more than 900 votes only in LIHC. C9 protein is a subunit of the complement membrane attack complex (MAC) that targets pathogen cell membranes to cause cell lysis and death in the immune system. TNF has been observed to be able to up-regulate the expression of *C9* [74], revealing its involvement in the NFκB/TNF pathway. Furthermore, G6PC has been reported to be up-regulated by HNF4A [75]. HNF4A is down-regulated by TNF [76] and suppressed by NFκB [77]. Additionally, mRNA expression of Tnf-α is significantly increased in G6pc knock-out mice [78]. These observations suggest a regulatory relationship between TNF and *G6PC*, although direct experimental evidence remains to be provided. It is worth noting that *C9* and *G6PC* have been reported to be highly and specifically expressed in liver tissue according to Genotype-Tissue Expression (GTEx, dbGaP, phs000424.v8.p2) and BioGPS [79]. Previous studies have uncovered the roles of *C9* and *G6PC* in the NFκB/TNF pathways and their tissue specificity in the liver. However, their connections to LIHC are not widely reported, necessitating further evidence to confirm this relationship.

On the other hand, candidate genes that receive votes in only one cancer type tend to have relatively low vote counts, with a median vote of 7 and a mean of 67.68. Most of these predictions (votes) may be attributed to random sampling. Accordingly, it is essential to exercise caution when evaluating the confidence of these potential cancer-specific candidates with low votes and low cancer prevalence in only one cancer type, and further investigation is required to confirm their association with specific cancer.

Although our ensemble learning model demonstrates exceptional performance overall, its prediction accuracy is relatively poor for KICH compared to the other 15 cancer types, with an AUC of 0.57 (Fig. 1c). Likewise, the GSEA analysis of cancer-dysregulated genes in KICH showed overrepresentation as well, though only with moderate significance (Fig. 3a and Additional file 1: Fig. S3). In addition, the identified highly-voted functional modules in most cancer types encompassed a wide range of biological processes, including regulation of tumor necrosis factor production, cellular response to cytokine stimulus, adaptive immunity, anti-apoptosis, cell migration, and angiogenesis (Fig. 2b). These functional modules are closely related to the canonical NFκB-regulated pathway and cancer progression. However, in the case of KICH, the identified functional modules are associated with immunity only, lacking the presence of other types of functional modules. This observation suggests that the mechanism of the NFκB/TNF pathway in KICH may differ from other cancer types.

Besides investigating TNBC, we analyzed the relationship between smoking history and patients’ coefficients in the LUAD ensemble model. Previous studies have reported that tobacco smoking could induce the inflammation associated with the TNF and NFκB signaling pathways, promoting the development of lung cancer [80, 81]. We found that non-smoking patients tended to possess smaller coefficients with moderate significance (z-score = − 1.25, Additional file 1: Fig. S10a), suggesting that non-smoking patients might be less affected by the NFκB/TNF pathway. Conversely, smoking patients, including reformed smokers, tended to have larger coefficients (Additional file 1: Fig. S10b), although this association is not statistically significant (z-score = 0.83). Notably, most patients in the LUAD dataset are smokers (433/508 = 85%), which may have influenced the insignificant association between the coefficients and smoking history. Nevertheless, our study still confirmed a positive correlation. Combined with the TNBC analysis, the findings from the LUAD patients reinforce the capability of our ensemble model in identifying patients with specific cancer types associated with the NFκB/TNF-regulated pathways.

## Conclusions

We have developed an ensemble learning model that can predict genes involved in the NFκB/TNF-regulated pathways and carcinogenesis. This approach differs from the existing prediction models by utilizing gene expression profiles of cancer patients as features and genes as samples. The genes identified by the model are likely involved in the NFκB/TNF-regulated pathways, particularly the canonical ones, and exert a significant influence on cancer progression and patient prognosis. Importantly, our model offers interpretability, enabling the identification of specific cancer subtypes, such as TNBC. Additionally, we have demonstrated that the functional module of mononuclear cell differentiation can accurately predict the TNBC status and assess the prognosis of non-TNBC patients. This ensemble learning model demonstrates exceptional predictive performance in identifying genes associated with specific mechanisms and effectively handles highly imbalanced data. By providing precise targets for precision medicine in cancer subtypes, our model could offer valuable insights for tailored treatment approaches. Moreover, its flexibility, traceability, retrospective capability, and interpretability enhance its utility in cancer research.

## Supplementary Information

**Additional file 1: Figure S1**: The association between the cancer prevalence and the percentage of NFκB/TNF hallmark genes. The cancer prevalence of a gene is the number of cancers that vote for the gene. The percentage on the y-axis is the proportion of NFκB/TNF hallmark genes under the corresponding cancer prevalence. The odds ratio is the ratio of the odds of the NFκB/TNF hallmark genes voted by the number of cancer types on the x-axis to the odds of the non-NFκB/TNF hallmark genes voted by the number of cancer types on the x-axis. Green and red circles show that the NFκB/TNF hallmark gene proportions under the corresponding cancer prevalence are significantly underrepresented and overrepresented, respectively. Grey circles represent insignificance. The significance is determined by Fisher’s exact test with a *p*-value < 0.05. **Figure S2**: Enrichment analysis of the functional similarity between candidates from the conventional approach and core member genes. **Figure S3**: Enrichment score curve of cancer-dysregulated genes based on votes. Gene set enrichment analysis (GSEA) was conducted to test the enrichment of the cancer-dysregulated genes from the C6 gene set of MSigDB on genes' votes. The x-axis represents the voted genes arranged in descending order of their votes and average votes in each cancer type and pan-cancer model, respectively. The red dot indicates the maximum enrichment score (ES). The dashed line represents the maximum ES, while the dotted line represents the baseline of zero. The *p*-values and z-scores of the ES were assessed by 10,000 random sampling processes. **Figure S4**: Proportion of cancer-dysregulated (C6) genes across vote thresholds in each cancer and pan-cancer model. **Figure S5**: Enrichment score curve of cancer-dysregulated (C6) genes on correlation. **Figure S6**: Proportion of cancer-dysregulated (C6) genes across association score thresholds in each cancer and pan-cancer model. **Figure S7**: Enrichment analysis of BRCA subtypes. **Figure S8**: 3-year hazard ratios of genes in the functional module mononuclear cell differentiation for non-TNBC patients. **Figure S9**: Consistency of gene votes between ensemble learning model with 500 and 1000 member classifiers in 16 cancer types. **Figure S10**: Enrichment analysis of LUAD subtypes based on smoking history. **Table S1**. Count of gene categories in the ensemble learning models. **Table S2**: The biological processes with long descriptions in Figure 2. **Table S3**: The identified highly-voted functional modules activated/inactivated in TNBC patients.

## Data Availability

All data used could be obtained from public sources (details in Supplementary Information: SI Materials and Methods). The mRNA expression profiles (RNA-Seq data) of studied cancer types were obtained from the TCGA via the Genomic Data Commons Portal: https://portal.gdc.cancer.gov/. The two external mRNA expression profiles of breast cancer (E-GEOD-58135 and E-GEOD-76250) were downloaded from ArraryExpress. The hallmark gene set of NFκB/TNF regulatory pathway (HALLMARK_TNFA_SIGNALING_VIA_NFKB) and cancer-dysregulated gene set (C6) were downloaded from MSigDB. The functional annotation of genes was downloaded from the Gene Ontology (GO) database. The human protein–protein interaction (PPI) data was obtained from the InBio Map database. The implementation of the proposed ensemble model, processed data, and parsed results are available at https://github.com/yinyuansu/NFkB_TNFa_HM.

## Abbreviations

NFκB: Nuclear factor kappa B
TNF: Tumor necrosis factor
TNBC: Triple-negative breast cancer
SVM: Support vector machine
AUC: Area under the receiver operating characteristic curve
ROC: Receiver operating characteristic
GSEA: Gene set enrichment analysis
BLCA: Bladder urothelial carcinoma
BRCA: Breast cancer
CESC: Cervical squamous cell carcinoma and endocervical adenocarcinoma
CHOL: Cholangiocarcinoma
COAD: Colon adenocarcinoma
ESCA: Esophageal carcinoma
GBM: Glioblastoma multiforme
HNSC: Head and neck squamous cell carcinoma
KICH: Chromophobe renal cell carcinoma
KIRC: Clear cell kidney carcinoma
KIRP: Papillary kidney carcinoma
LGG: Brain lower grade glioma
LIHC: Liver hepatocellular carcinoma
LUAD: Lung adenocarcinoma
LUSC: Lung squamous cell carcinoma
OV: Ovarian serous cystadenocarcinoma
PAAD: Pancreatic adenocarcinoma
PRAD: Prostate adenocarcinoma
SARC: Sarcoma
STAD: Stomach adenocarcinoma
THCA: Papillary thyroid carcinoma
UCEC: Uterine corpus endometrial carcinoma
ER: Estrogen receptor
PR: Progesterone receptor
HER2: Human epidermal growth factor receptor 2
AUCPR: Area under the precision-recall curve
PERMANOVA: Permutational multivariate analysis of variance
