# Supplementary material for: Ensemble learning model for identifying the hallmark genes of NFκB/TNF signaling pathway in cancers
Source: J Transl Med. 2023 Jul 20;21:485. doi: 10.1186/s12967-023-04355-5 (PMC10357720; doi:10.1186/s12967-023-04355-5)
Supplement: Supplementary file 1 — Additional file 1: Figure S1: The association between the cancer prevalence and the percentage of NFκB/TNF hallmark genes. The cancer prevalence of a gene is the number of cancers that vote for the gene. The percentage on the y-axis is the proportion of NFκB/TNF hallmark genes under the corresponding cancer prevalence. The odds ratio is the ratio of the odds of the NFκB/TNF hallmark genes voted by the number of cancer types on the x-axis to the odds of the non-NFκB/TNF hallmark genes voted by the number of cancer types on the x-axis. Green and red circles show that the NFκB/TNF hallmark gene proportions under the corresponding cancer prevalence are significantly underrepresented and overrepresented, respectively. Grey circles represent insignificance. The significance is determined by Fisher’s exact test with a p-value < 0.05. Figure S2: Enrichment analysis of the functional similarity between candidates from the conventional approach and core member genes. Figure S3: Enrichment score curve of cancer-dysregulated genes based on votes. Gene set enrichment analysis (GSEA) was conducted to test the enrichment of the cancer-dysregulated genes from the C6 gene set of MSigDB on genes' votes. The x-axis represents the voted genes arranged in descending order of their votes and average votes in each cancer type and pan-cancer model, respectively. The red dot indicates the maximum enrichment score (ES). The dashed line represents the maximum ES, while the dotted line represents the baseline of zero. The p-values and z-scores of the ES were assessed by 10,000 random sampling processes. Figure S4: Proportion of cancer-dysregulated (C6) genes across vote thresholds in each cancer and pan-cancer model. Figure S5: Enrichment score curve of cancer-dysregulated (C6) genes on correlation. Figure S6: Proportion of cancer-dysregulated (C6) genes across association score thresholds in each cancer and pan-cancer model. Figure S7: Enrichment analysis of BRCA subtypes. Figure S8: 3-year h [file 12967_2023_4355_MOESM1_ESM.pdf]

Additional file

Ensemble learning model for identifying the hallmark genes of NFκB/TNF signaling pathway in cancers

Yin-Yuan Su, Yu-Ling Liu, Hsuan-Cheng Huang, Chen-Ching Lin

Contents

SI Materials and Methods 2
mRNA expression profiles in cancers 2
Hallmark genes and cancer-dysregulated genes (C6) in MSigDB 2
Functional similarity analysis of the identified candidate genes 3
Identification of highly-voted functional modules within the candidates 3
Enrichment analysis of gene sets and patient groups 5
Survival analysis of the identified genes 6
Classification of breast cancer subtypes 6
Prediction model in classifying patients' TNBC status 7
The conventional approach for identification of the genes of interest 7
SI Figures 9
Figure S1: The association between the cancer prevalence and the percentage of NFκB/TNF hallmark genes. 9
Figure S2: Enrichment analysis of the functional similarity between candidates from the conventional approach and core member genes. 9
Figure S3: Enrichment score curve of cancer-dysregulated genes based on votes. 10
Figure S4: Proportion of cancer-dysregulated (C6) genes across vote thresholds in each cancer and pan-cancer model. 11
Figure S5: Enrichment score curve of cancer-dysregulated (C6) genes on correlation. 12
Figure S6: Proportion of cancer-dysregulated (C6) genes across association score thresholds in each cancer and pan-cancer model. 13
Figure S7: Enrichment analysis of BRCA subtypes 14
Figure S8: 3-year hazard ratios of genes in the functional module mononuclear cell differentiation for non-TNBC patients. 15
Figure S9: Consistency of gene votes between ensemble learning model with 500 and 1000 member classifiers in 16 cancer types. 16
Figure S10: Enrichment analysis of LUAD subtypes based on smoking history 16
SI Tables 17
Table S1. Count of voted genes in the ensemble learning models 17
Table S2: The biological processes with long descriptions in Figure 2. 18
Table S3: The identified highly-voted functional modules activated/inactivated in TNBC patients. 19
References 20

SI Materials and Methods

mRNA expression profiles in cancers

The mRNA expression profiles of sixteen cancer types: bladder urothelial carcinoma (BLCA), breast cancer (BRCA), cholangiocarcinoma (CHOL), colon adenocarcinoma (COAD), esophageal carcinoma (ESCA), head and neck squamous cell carcinoma (HNSC), chromophobe renal cell carcinoma (KICH), clear cell kidney carcinoma (KIRC), papillary kidney carcinoma (KIRP), liver hepatocellular carcinoma (LIHC), lung adenocarcinoma (LUAD), lung squamous cell carcinoma (LUSC), prostate adenocarcinoma (PRAD), stomach adenocarcinoma (STAD), papillary thyroid carcinoma (THCA), and uterine corpus endometrial carcinoma (UCEC) were obtained from the RNA-Seq data in The Cancer Genome Atlas (TCGA). RNA-Seq data from every patient contain 19,671 protein-coding genes. This number is the same for all cancer types. We used the upper-quartile normalized read counts inferred via RSEM (RNA-Seq by Expectation Maximization) [1] algorithm from RNA-Seq data as mRNA expression level. Furthermore, we performed standardization (z-score normalization) for feature rescaling to eliminate the analysis error caused by the difference among patients. Since the patients are features, the feature standardization transforms the distribution of each patient's gene expression level with zero mean and unit variance. It was calculated as follows:

$$x' = \frac{x - \bar{x}}{\sigma}$$

where x and x' are the original and standardized values of gene expression level, respectively; $\bar{x}$ is the mean of the patient's gene expression level, and σ is the standard deviation. The two external mRNA expression profiles of breast cancer (E-GEOD-58135 [2] and E-GEOD-76250 [3]) that were used to validate the capability of the identified functional module in predicting TNBC were downloaded from the ArrayExpress.

Hallmark genes and cancer-dysregulated genes (C6) in MSigDB

During model training, we used the "HALLMARK_TNFA_SIGNALING_VIA_NFKB" hallmark gene set (abbreviated as NFκB/TNF hallmark genes) from MSigDB [4], which covers 200 genes, as the positive data (sample). Out of the 200 genes in this gene set, the TCGA RNA-Seq data covers 198 genes. In addition, there are 4,304 genes covered by other hallmark gene sets in MSigDB. Of these, 157 genes are also included in the

NFκB/TNF hallmark gene set, and other hallmark genes cover the remaining 4,147 genes. We excluded these 4,147 genes from the training stages to avoid potential disturbances during model learning, as they may be potentially associated with cancer progression. We then applied the trained ensemble learning model to the complete genome data, including the 4,147 genes that were previously excluded, to predict the potential cancer-influential genes involved in the NFκB/TNF pathway. To further investigate the oncogenicity of the predicted candidates that are not NFκB/TNF hallmark genes and have at least one vote across all cancer types, we obtained the oncogenic gene set (C6) from MSigDB. The C6 gene set consists of 189 gene sets, totaling 12,493 genes, and encompasses genes that are dysregulated in cancers by affecting known cancer genes.

Functional similarity analysis of the identified candidate genes

We downloaded functional annotation data of genes from the Gene Ontology (GO) database. In this study, we only used the biological processes domain. We used the Jaccard index, calculated from the shared experimentally validated biological processes, to evaluate the functional similarity between each gene in the RNA-Seq data and the six core member genes of the TNF and NFκB family genes. Notably, only the experimentally validated biological processes with a depth smaller than seven in the GO database were used to calculate the Jaccard index similarity. In the GO tree structure, 'depth' is defined as the shortest path length from a GO term to the root term, in this case, the 'biological process' (GO:0008150). To determine if the candidate genes significantly participate in the NFκB-regulated pathways downstream of TNF, we employed gene set enrichment analysis (GSEA). We further excluded genes with zero similarity to the corresponding core member gene to ensure a more representative trend. The similarities between NFκB/TNF core members and all genes were ranked based on the functional similarity score to hit the identified candidate genes. The significance of the enrichment score was assessed by 10,000 permutation tests.

Identification of highly-voted functional modules within the candidates

To interrogate the biological processes in which the candidate genes participate during carcinogenesis, we performed conventional and network-wise functional enrichment analyses combined with GSEA to identify the highly-voted functional modules for each cancer type. In the conventional functional enrichment analysis, the significance was established by the p-value derived from the hypergeometric test, which is described below:

$$P(X = k) = \frac{\binom{m}{k}\binom{N-m}{n-k}}{\binom{N}{n}}$$

where X denotes the evaluated function; N represents the number of GO annotated genes; m indicates the number of candidates; n represents the number of genes with the evaluated function, and k indicates the number of candidates with the evaluated function.

We also incorporated protein interaction and network-wise functional enrichment analyses to enhance the functional relationship between the identified candidates to discover the functional modules formed by candidates [5]. The human protein-protein interaction (PPI) data source was obtained from the InBio Map database [6]. The network-wise functional enrichment analysis was modified from the conventional method. The test function's significance was based on p-values produced from a modified hypergeometric test. The hypergeometric distribution for the network-wise approach is described below:

$$P_e(X = k_e) = \frac{\binom{m_e}{k_e}\binom{N_e-m_e}{n_e-k_e}}{\binom{N_e}{n_e}}$$

where e is the abbreviation of the PPI. Each symbol has the same meaning as the conventional hypergeometric distribution, but the counting objects are changed from genes to functional PPIs. The functional PPIs are interactions formed by the two genes involved in the same functions. This approach revealed the significant protein interaction functional modules in which the identified candidates were involved. The two p-values from the conventional and network-wise analysis were adjusted by the Benjamini and Hochberg multiple testing procedures to control the false discovery rate (FDR) [7]. Subsequently, the functional modules with adjusted p-value < 0.05 from both analyses were identified as significantly enriched functional modules. Additionally, only the significantly enriched functional modules with a depth greater than six in the GO database were used to increase the functional specificity.

Next, we applied GSEA to select the significantly candidate-enriched functional modules that also significantly overrepresent the highly voted genes. Herein, we performed GSEA by ranking the candidates based on their votes in the corresponding cancer type to hit the genes in the gene set of the tested functional module to

calculate the enrichment score. The significance of the enrichment score, i.e., z-score, was assessed by 1,000 permutation tests. The functional modules with z-score ≧ 2 were denoted as the highly-voted candidate-enriched functional modules. In a word, the final identified functional modules are formed by the highly voted candidates involved in the same functions, as well as forming the PPI among them. The discovered significantly highly-voted candidate-enriched functional modules were further summarized by the REVIGO [8] algorithm, with a similarity ≥0.9, which was calculated using the Resink algorithm[9], and visualized using the CirGO package [10].

Enrichment analysis of gene sets and patient groups

In this study, we performed GSEA in the functional similarity analysis, identifying highly-voted candidate-enriched functional modules, investigating the identified genes' oncogenicity and poor-prognostic characteristics, and assessing the activity of the highly voted functional modules identified in TNBC analysis. In functional similarity analysis, the similarities between NFκB/TNF core members and genes were ranked to hit the identified candidate genes; in the identification of highly-voted candidate-enriched functional modules, the votes of candidates were ranked to hit candidates in the functional modules; in the investigation of identified genes' oncogenicity and poor-prognostic characteristics, the votes of identified genes were ranked to hit cancer-dysregulated genes (C6) and poor-prognostic genes, respectively [11]; in assessing the activity of the modules identified in TNBC analysis, the fold-change of gene expression level were ranked to hit the member genes in the module. The fold change of each gene was calculated from the differential expression analysis between TNBC and non-TNBC implemented by the Limma-Voom R package [13]. Patient set enrichment analysis (PSEA) was performed to study the association between patients' contributions (magnitude of coefficients in the model) to the ensemble model and cancer subtypes. That is, we applied the concept of GSEA to test the correlation between patients' average absolute weight across 1,000 member classifiers and TNBC status, as well as the status of the receptors－estrogen receptor (ER), progesterone receptor (PR), human epidermal growth factor receptor 2 (HER2). More specifically, we tested if patients with higher absolute weight tend to be with ER-, PR-, HER2-, or TNBC. In PSEA, the average absolute weight of each patient in the ensemble model was ranked to hit patients with the investigated subtype. All enrichment analyses used descending rank and unweighted enrichment score

(ES) for hits. The significance of the enrichment score, i.e., z-score, was assessed by 10 thousand permutation tests.

Survival analysis of the identified genes

To investigate the impact of the identified (voted) genes on patient survival, we collected a pan-cancer survival influential gene set from our previous study [11]. This pan-cancer survival influential gene set is identified by the Cox-regression model [12]. Specifically, that study developed a systematic process to determine the most appropriate cut-off of significance level for identifying survival influential genes across 16 cancer types: BLCA, BRCA, COAD, ESCA, HNSC, KIRC, LIHC, LUAD, LUSC, STAD, CESC, GBM, LGG, OV, PAAD, and SARC. The first ten cancer types are also in our ensemble model and referred to as the internal data set. In contrast, the remaining 6 cancers were not used during the model training and designated as the independent data set. By using two separate data sets, we can ensure that the model does not overfit the training data and can accurately predict patient survival for all cancers. Among the 4,678 identified genes in the pan-cancer model, 4,022 are provided with a pre-calculated hazard ratio (the exponential regression coefficient) in the Cox regression model from our previous study. We then calculated patients' risk scores to determine the impact of the identified genes on patient outcomes. For each patient, the risk score is defined below:

$$risk\ score = \sum_{i=1}^{n} \frac{\theta_i * e_i}{n}$$

where n is the number of identified genes, e_i represents the standardized expression level of gene i, and θ_i denotes the coefficient of gene i calculated from the Cox regression model in our previous study [11]. Subsequently, we stratified patients into two groups, namely high-risk and low-risk, using the median risk score as the cut-off. The prognostic effect and significance of the identified genes were evaluated using Kaplan-Meier plots and the log-rank test.

Classification of breast cancer subtypes

We classified the breast cancer patients into subtypes according to their receptor (ER and HER2) status. First, the patients who are recorded to have three receptors (ER, PR, and HER2) all negative are classified as TNBC type; the other patients are denoted as non-TNBC type, and further classified as types of luminal, LumA, LumB,

HER2+, and unclassified. The patients who express ER were first classified as luminal type. The patients of luminal type were then subclassified into LumA (not express HER2) and LumB (express HER2). The patients who do not express ER but express HER2 were classified as HER2+. Finally, the patients who did not belong to the above subtypes (TNBC, luminal, LumA, LumB, HER2+) were categorized as unclassified type.

Prediction model in classifying patients' TNBC status

To test the performance of the identified functional modules in predicting TNBC patients, we trained a logistic regression model for each module using gene expression profiles in the corresponding module. For each module, we performed 100 hold-out processes to assess the potential overfitting. During the hold-out process, 60% of the sampling data was used for training, and the remaining 40% was used for testing. The performance was then evaluated by AUC and AUCPR values derived from the testing data. On the other hand, we merged three datasets, which are TCGA, E-GEOD-58135 [2], and E-GEOD-76250 [3], to train a global model (logistic regression model) to estimate the contribution of each gene in the functional module of mononuclear cell differentiation in predicting TNBC patients. The contribution of each gene was then evaluated by the effect size, which is the z-score estimated by Wald's test, in the model.

The conventional approach for identification of the genes of interest

To demonstrate the performance of our ensemble learning model, we performed the conventional approach that used the Pearson correlation coefficient (PCC) of gene expression to identify the genes highly associated with the 198 NFκB/TNF hallmark genes in carcinogenesis. We first calculated the PCC between each gene and the 198 NFκB/TNF hallmark genes for each cancer type. Then, for each gene, the median of the absolute values of these 198 PCCs was used as its association score with the 198 NFκB/TNF hallmark genes in the corresponding cancer type. In pan-cancer analysis, the mean value of the association score was adopted. We calculated the AUC derived from the association score to predict the NFκB/TNF hallmark genes to evaluate the prediction accuracy. For the enrichment analysis in oncogenicity and functional similarity with NFκB/TNF core members, the top *n* genes with high association scores were denoted as the conventional approach's candidates. The value of *n* is the number of voted genes identified by our ensemble learning approach in the corresponding cancer type or pan-cancer analysis. Then, the same procedure of enrichment analysis in oncogenicity and functional similarity with

NFκB/TNF core members was applied to the candidates identified by the conventional approach.

SI Figures

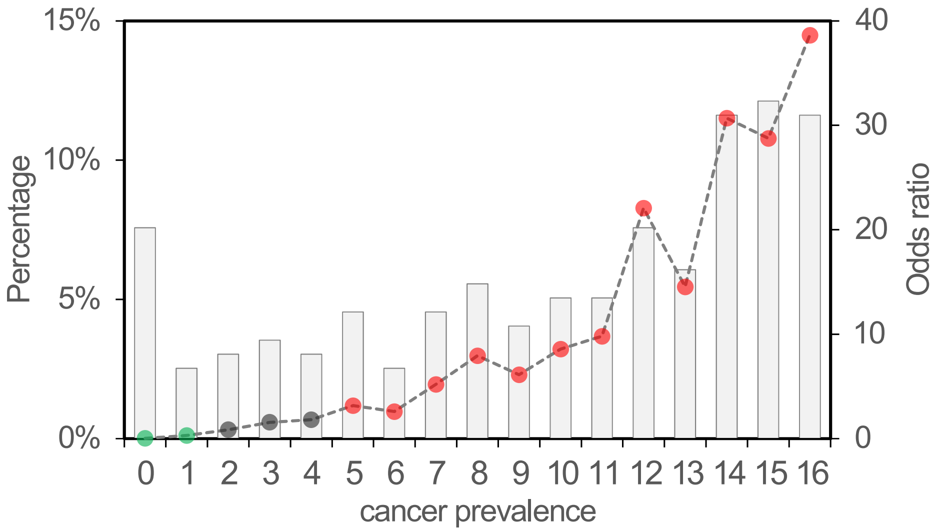

Figure S1: The association between the cancer prevalence and the percentage of NFκB/TNF hallmark genes. The cancer prevalence of a gene is the number of cancers that vote for the gene. The percentage on the y-axis is the proportion of NFκB/TNF hallmark genes under the corresponding cancer prevalence. The odds ratio is the ratio of the odds of the NFκB/TNF hallmark genes voted by the number of cancer types on the x-axis to the odds of the non-NFκB/TNF hallmark genes voted by the number of cancer types on the x-axis. Green and red circles show that the NFκB/TNF hallmark gene proportions under the corresponding cancer prevalence are significantly underrepresented and overrepresented, respectively. Grey circles represent insignificance. The significance is determined by Fisher's exact test with a *p*-value < 0.05.

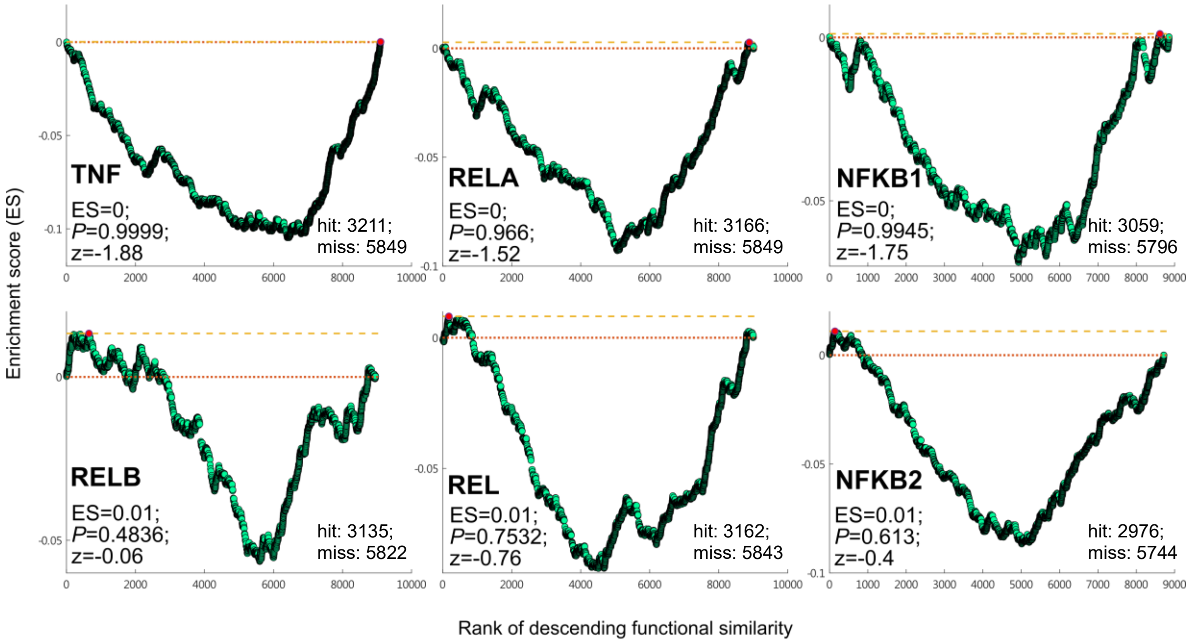

Figure S2: Enrichment analysis of the functional similarity between candidates from the conventional approach and core member genes.

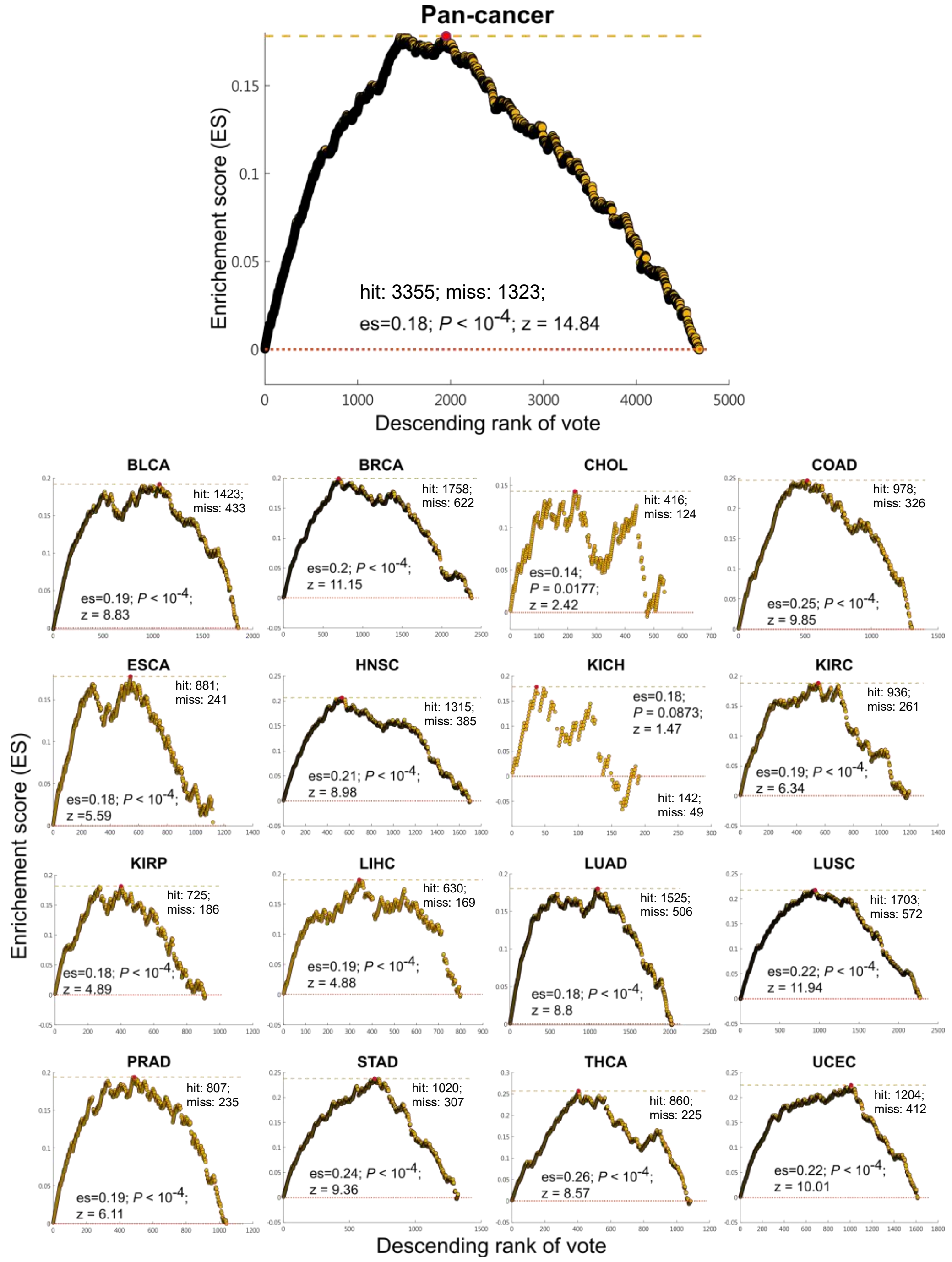

Figure S3: Enrichment score curve of cancer-dysregulated genes based on votes.

Gene set enrichment analysis (GSEA) was conducted to test the enrichment of the cancer-dysregulated genes from the C6 gene set of MSigDB on genes' votes. The x-axis represents the voted genes arranged in descending order of their votes and average votes in each cancer type and pan-cancer model, respectively. The red dot indicates the maximum enrichment score (ES). The dashed line represents the maximum ES, while the dotted line represents the baseline of zero. The *p*-values and z-scores of the ES were assessed by 10,000 random sampling processes.

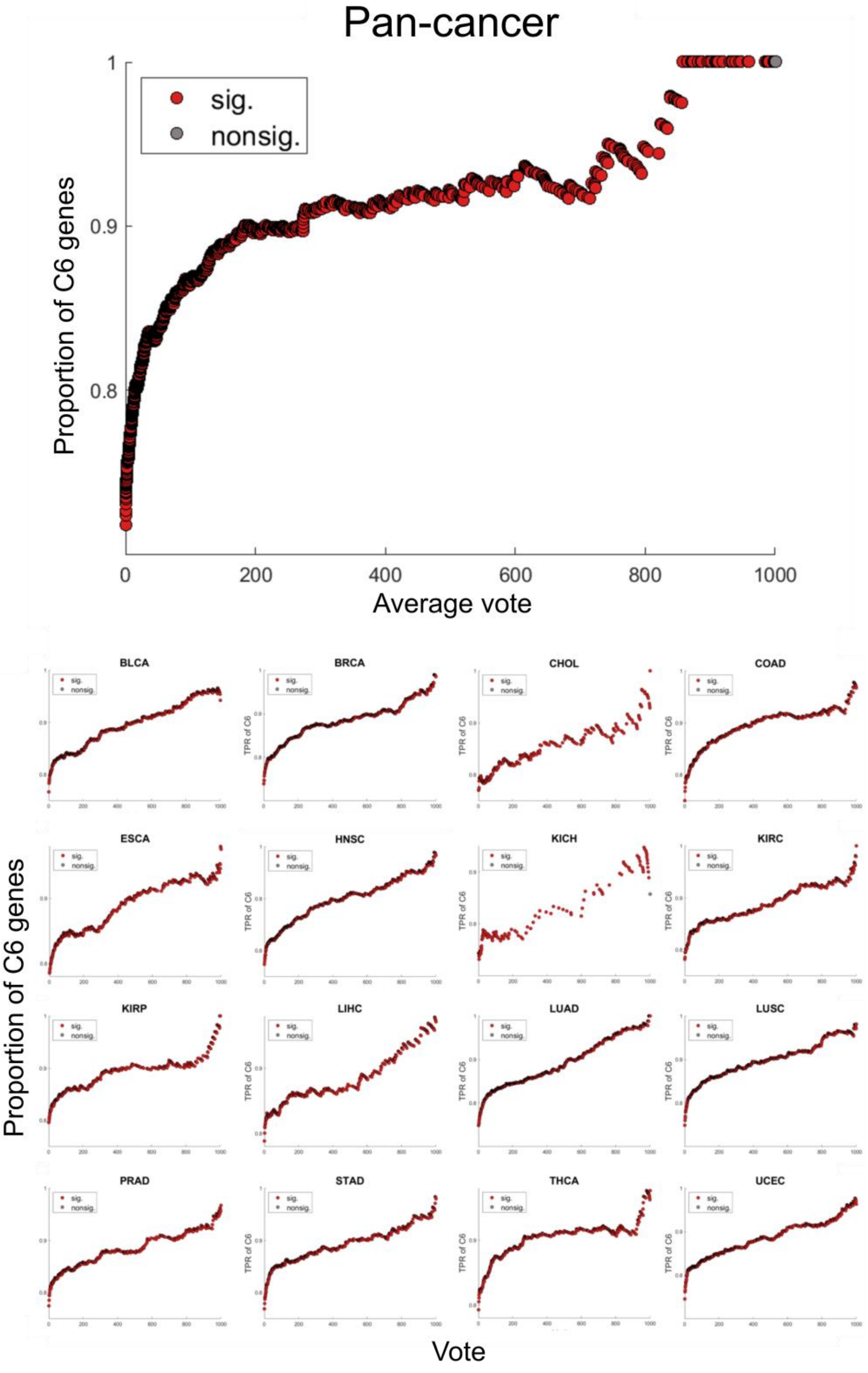

Figure S4: Proportion of cancer-dysregulated (C6) genes across vote thresholds in each cancer and pan-cancer model.

Each dot on the curve represents the proportion of cancer-dysregulated genes in the category with votes no less than the threshold indicated on the x-axis. Red dots indicate a significant enrichment determined by Fisher's exact test, while gray dots indicate insignificance.

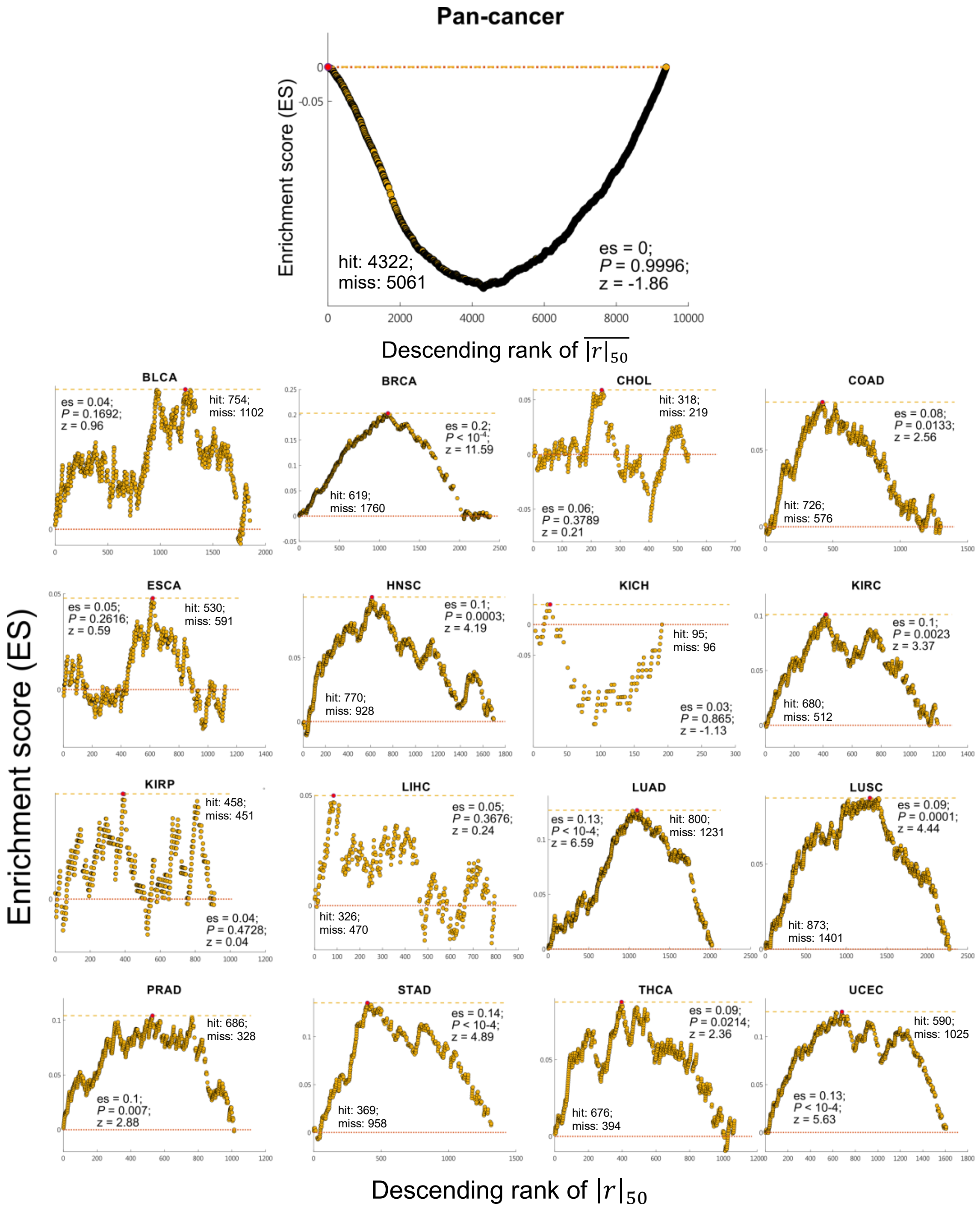

Figure S5: Enrichment score curve of cancer-dysregulated (C6) genes on correlation.

GSEA was conducted to test the enrichment of the cancer-dysregulated genes from the C6 gene set of MSigDB on genes' association scores with 198 NFκB/TNF hallmark genes. The association score, the label of the x-axis $|r|_{50}$, for individual cancer type is the median of absolute PCC with 198 NFκB/TNF hallmark genes. The pan-cancer association score, the label of the x-axis $\overline{|r|_{50}}$, is the mean of the association scores (median absolute PCC with 198 NFκB/TNF hallmark genes) across 16 cancer types. The dashed line represents the maximum ES, while the dotted line represents the baseline of zero. The *p*-values and z-scores of the ES were assessed by 10,000 random sampling processes.

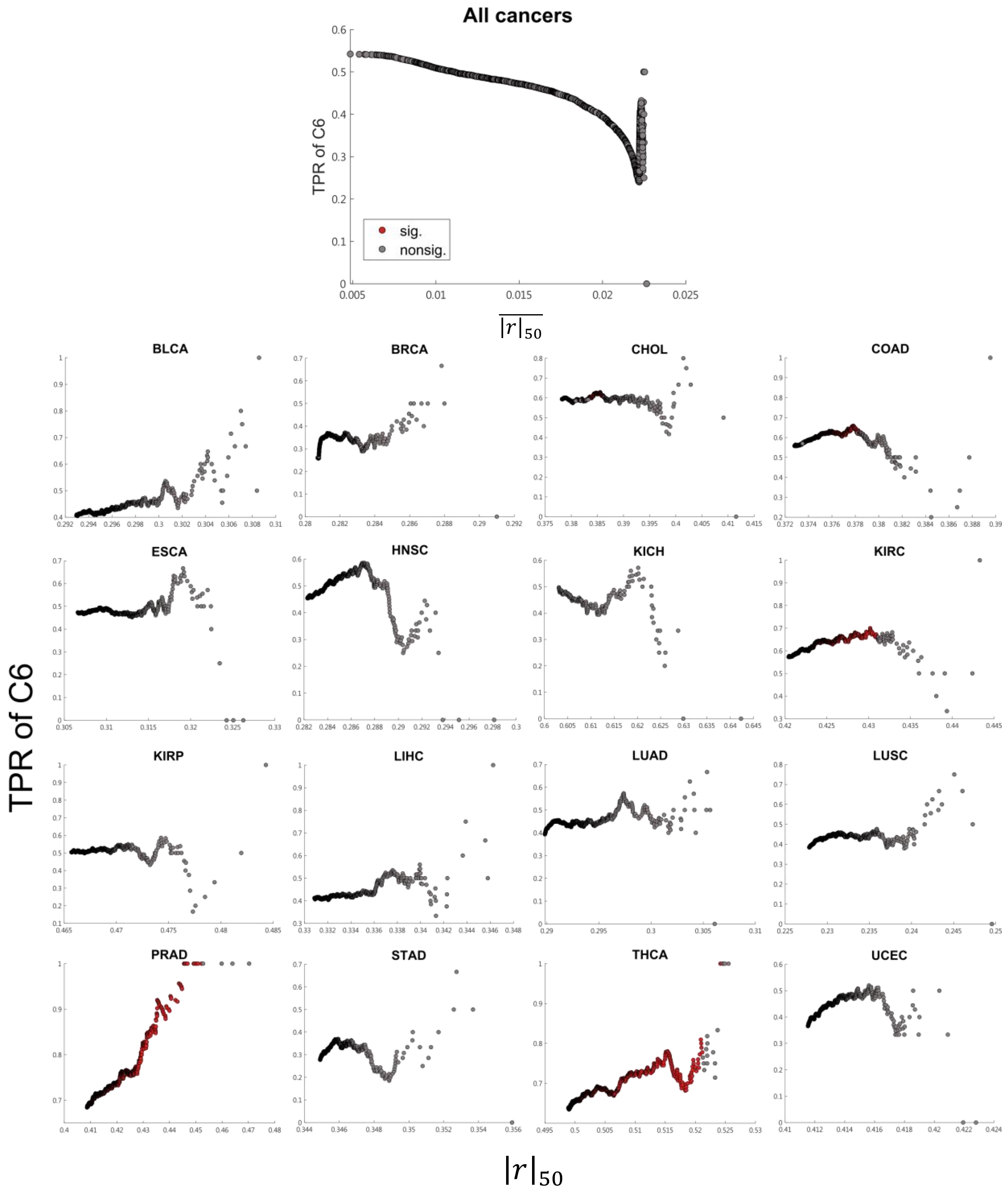

Figure S6: Proportion of cancer-dysregulated (C6) genes across association score thresholds in each cancer and pan-cancer model.

Each dot on the curve represents the proportion of cancer-dysregulated genes in the category with an association score no less than the threshold indicated on the x-axis. The association score, the label of the x-axis $|r|_{50}$, for individual cancer type is the median of absolute PCC with 198 NFκB/TNF hallmark genes. The pan-cancer association score, the label of the x-axis $\overline{|r|_{50}}$, is the mean of the association scores (median absolute PCC with 198 NFκB/TNF hallmark genes) across 16 cancer types. Red dots indicate a significant enrichment determined by Fisher's exact test, while gray dots indicate insignificance.

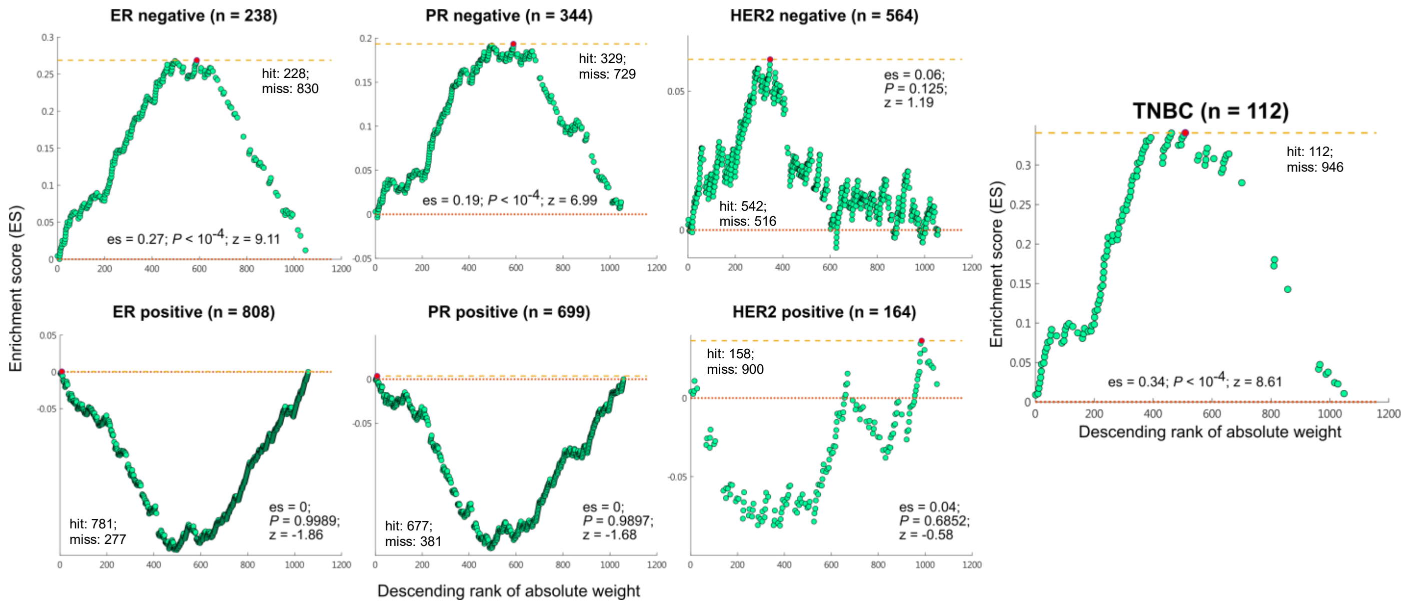

Figure S7: Enrichment analysis of BRCA subtypes

PSEA was conducted to assess the enrichment of different BRCA subtypes. The BRCA subtypes were classified based on receptor status, including ER +/-, PR+/-, HER2+/-, and triple-negative (ER-, PR-, HER2-). The x-axis represents the decreasing rank of the absolute weights (coefficients) of the 1,000 member classifiers for each BRCA patient. Each green dot on the curve is the ES of the hit patients with the corresponding BRCA subtype. The red dot represents the maximum ES. The dashed line represents the maximum ES, while the dotted line represents the baseline of zero ES. The significance of the enrichment score was assessed using 10,000 permutation tests. *n* represents the number of patients with the corresponding BRCA subtype in TCGA, whereas "hit" represents the subset of these patients for whom RNA-Seq data is available.

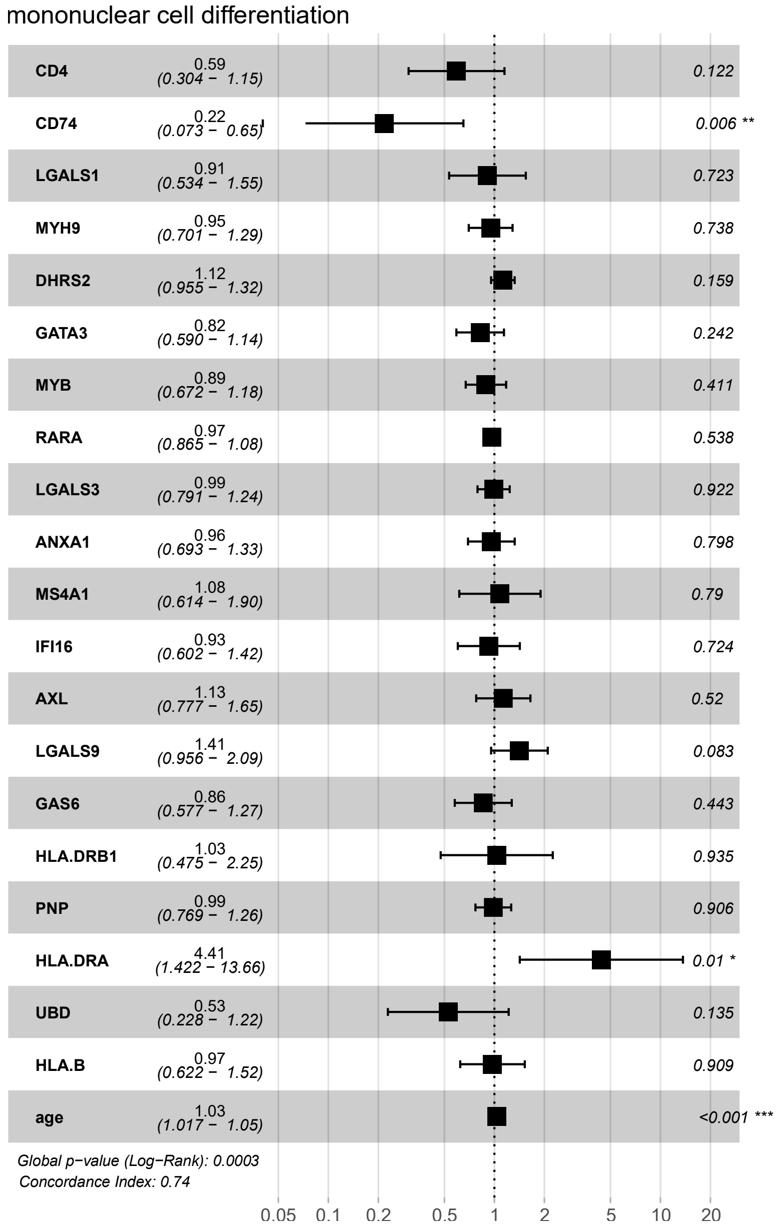

Figure S8: 3-year hazard ratios of genes in the functional module mononuclear cell differentiation for non-TNBC patients.

The hazard ratios were calculated by a multivariate Cox regression model using age and expression profiles of genes in the functional module of mononuclear cell differentiation as covariates. The forest plot shows the hazard ratios and the corresponding confidence interval of each gene and age in the Cox regression model.

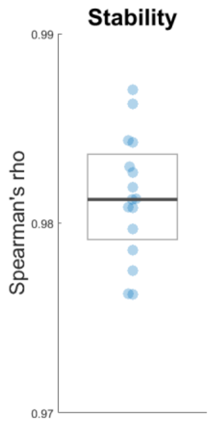

Figure S9: Consistency of gene votes between ensemble learning model with 500 and 1000 member classifiers in 16 cancer types.

The figure illustrates Spearman's ρ correlation coefficient of gene votes between ensemble models with 500 and 1,000 member classifiers for each cancer type. Genes with zero votes in both models were excluded from the calculation of Spearman's ρ. The obtained ρ values range from 0.97 to 0.99 across 16 cancers, indicating the high stability of our ensemble model. It also proves that running 1,000 times is sufficient to capture the overall information for the different cancer types.

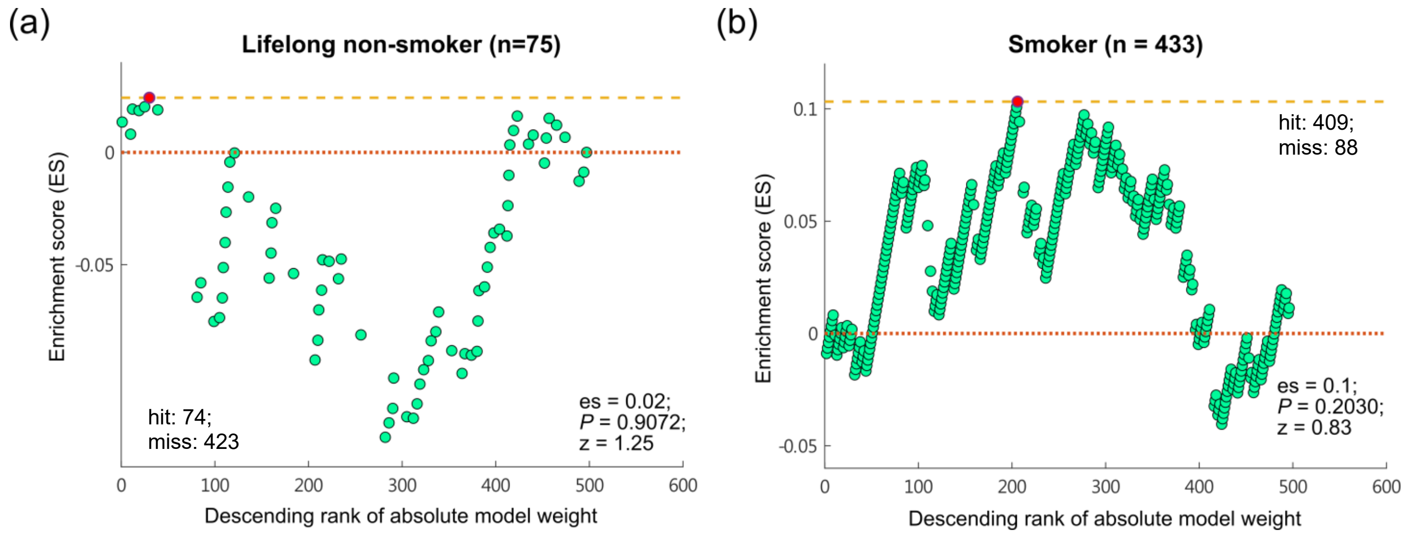

Figure S10: Enrichment analysis of LUAD subtypes based on smoking history

PSEA was conducted to examine different clinical statuses with LUAD subtypes based on the patient's smoking history. The LUAD subtypes were classified based on smoking history. The subtypes include (a) lifelong non-smokers (less than 100 cigarettes smoked in a lifetime); (b) smokers, comprising current smokers (including daily smokers and nondaily smokers or occasional smokers), current reformed smokers for more than 15 years, current reformed smokers for 15 years or less, and current reformed smokers, duration not specified. n represents the number of patients with the corresponding smoker type in TCGA, whereas "hit" represents the subset of these patients for whom RNA-Seq data is available.

SI Tables

Table S1. Count of gene categories in the ensemble learning models

cancer	voted gene[1]	candidate[2]	voted NFκB/TNF hallmark[3]	non-candidate[4]
pan-cancer	4678	4495	183	14993
brca	2380	2211	169	17291
lusc	2275	2104	171	17396
luad	2031	1870	161	17640
blca	1856	1698	158	17815
hnsc	1700	1535	165	17971
ucec	1616	1487	129	18055
stad	1327	1200	127	18344
coad	1304	1180	124	18367
kirc	1197	1069	128	18474
esca	1122	998	124	18549
thca	1085	968	117	18586
prad	1042	928	114	18629
kirp	911	805	106	18760
lihc	799	722	77	18872
chol	540	485	55	19131
kich	191	162	29	19480

1. The total number of genes voted in each cancer and pan-cancer model.
2. The term "candidate" refers to the non-NFκB/TNF hallmark genes that received at least one vote in corresponding cancer or pan-cancer model.
3. The term "voted NFκB/TNF hallmark" refers to the NFκB/TNF hallmark genes that received at least one vote in corresponding cancer or pan-cancer model.
4. The term "non-candidate" refers to those genes having zero votes in corresponding cancer or pan-cancer model.

Table S2: The biological processes with long descriptions in Figure 2.

GOID	Description
GO:0002824	positive regulation of adaptive immune response based on somatic recombination of immune receptors built from immunoglobulin superfamily domains
GO:0002822	regulation of adaptive immune response based on somatic recombination of immune receptors built from immunoglobulin superfamily domains
GO:0002486	antigen processing and presentation of endogenous peptide antigen via MHC class I via ER pathway, TAP-independent
GO:0002484	antigen processing and presentation of endogenous peptide antigen via MHC class I via ER pathway
GO:0043516	regulation of DNA damage response signal transduction by p53 class mediator
GO:1903557	positive regulation of tumor necrosis factor superfamily cytokine production
GO:1902042	negative regulation of extrinsic apoptotic signaling pathway via death domain receptors
GO:1902041	regulation of extrinsic apoptotic signaling pathway via death domain receptors
GO:0008625	extrinsic apoptotic signaling pathway via death domain receptors
GO:2000446	regulation of macrophage migration inhibitory factor signaling pathway
GO:2000448	positive regulation of macrophage migration inhibitory factor signaling pathway
GO:0043618	regulation of transcription from RNA polymerase II promoter in response to stress
GO:1990440	positive regulation of transcription from RNA polymerase II promoter in response to endoplasmic reticulum stress
GO:1903321	negative regulation of protein modification by small protein conjugation or removal
GO:1903557	positive regulation of tumor necrosis factor superfamily cytokine production
GO:0008630	intrinsic apoptotic signaling pathway in response to DNA damage
GO:1902229	regulation of intrinsic apoptotic signaling pathway in response to DNA damage

Table S3: The identified highly-voted functional modules activated/inactivated in TNBC patients.

GOID	Description	N	E	Adj_Npva	Adj_Epva
GO:0070665	positive regulation of leukocyte proliferation	26	13	1.94E-02	3.90E-02
GO:0010628	positive regulation of gene expression	150	300	8.47E-06	1.01E-31
GO:0031328	positive regulation of cellular biosynthetic process	184	442	2.24E-02	2.23E-03
GO:0010557	positive regulation of macromolecule biosynthetic process	174	410	4.39E-02	3.19E-02
GO:0050863	regulation of T cell activation	39	18	1.89E-02	4.92E-02
GO:0031399	regulation of protein modification process	160	331	3.16E-03	1.36E-07
GO:0022409	positive regulation of cell-cell adhesion	49	24	1.53E-04	6.01E-03
GO:0071396	cellular response to lipid	61	41	6.34E-04	1.31E-03
GO:0071345	cellular response to cytokine stimulus	97	80	1.17E-09	3.66E-09
GO:0010629	negative regulation of gene expression	122	194	4.95E-05	3.44E-11
GO:0030335	positive regulation of cell migration	117	135	2.53E-18	2.60E-52
GO:1903131	mononuclear cell differentiation	28	20	1.39E-02	2.23E-07
GO:0002486	antigen processing and presentation of endogenous peptide antigen via MHC class I via ER pathway, TAP-independent	3	3	8.98E-03	2.58E-04
GO:0002484	antigen processing and presentation of endogenous peptide antigen via MHC class I via ER pathway	3	3	8.98E-03	2.58E-04
GO:0051248	negative regulation of protein metabolic process	133	196	9.95E-07	1.72E-18
GO:0007169	transmembrane receptor protein tyrosine kinase signaling pathway	65	119	2.90E-06	1.02E-35

1. N: number of genes in the functional module
2. E: number of protein interactions in the functional module
3. Adj_Npva: the adjusted *p*-value of the conventional functional enrichment analysis
4. Adj_Epva: the adjusted *p*-value of the network-wise functional enrichment analysis
5. The up-regulated and down-regulated functional modules are marked in red and green, respectively.

References

1. Li B, Dewey CN: **RSEM: accurate transcript quantification from RNA-Seq data with or without a reference genome**. *BMC Bioinformatics* 2011, **12**:323.
2. Varley KE, Gertz J, Roberts BS, Davis NS, Bowling KM, Kirby MK, Nesmith AS, Oliver PG, Grizzle WE, Forero A *et al*: **Recurrent read-through fusion transcripts in breast cancer**. *Breast Cancer Res Treat* 2014, **146**(2):287-297.
3. Liu YR, Jiang YZ, Xu XE, Hu X, Yu KD, Shao ZM: **Comprehensive Transcriptome Profiling Reveals Multigene Signatures in Triple-Negative Breast Cancer**. *Clin Cancer Res* 2016, **22**(7):1653-1662.
4. Liberzon A, Birger C, Thorvaldsdottir H, Ghandi M, Mesirov JP, Tamayo P: **The Molecular Signatures Database (MSigDB) hallmark gene set collection**. *Cell Syst* 2015, **1**(6):417-425.
5. Chen YR, Huang HC, Lin CC: **Regulatory feedback loops bridge the human gene regulatory network and regulate carcinogenesis**. *Brief Bioinform* 2019, **20**(3):976-984.
6. Li T, Wernersson R, Hansen RB, Horn H, Mercer J, Slodkowicz G, Workman CT, Rigina O, Rapacki K, Staerfeldt HH *et al*: **A scored human protein-protein interaction network to catalyze genomic interpretation**. *Nature methods* 2017, **14**(1):61-64.
7. Benjamini Y, Hochberg Y: **Controlling the false discovery rate: a practical and powerful approach to multiple testing**. *Journal of the Royal statistical society: series B (Methodological)* 1995, **57**(1):289-300.
8. Supek F, Bosnjak M, Skunca N, Smuc T: **REVIGO summarizes and visualizes long lists of gene ontology terms**. *PLoS One* 2011, **6**(7):e21800.
9. Resnik P: **Semantic similarity in a taxonomy: an information-based measure and its application to problems of ambiguity in natural language**. *J Artif Int Res* 1999, **11**(1):95–130.
10. Kuznetsova I, Lugmayr A, Siira SJ, Rackham O, Filipovska A: **CirGO: an alternative circular way of visualising gene ontology terms**. *BMC Bioinformatics* 2019, **20**(1):84.
11. Sahu D, Chang YL, Lin YC, Lin CC: **Characterization of the Survival Influential Genes in Carcinogenesis**. *Int J Mol Sci* 2021, **22**(9).
12. Cox DR: **Regression Models and Life-Tables**. *Journal of the Royal Statistical Society Series B (Methodological)* 1972, **34**(2):187-220.
13. Law CW, Chen Y, Shi W, Smyth GK: **voom: Precision weights unlock linear model analysis tools for RNA-seq read counts**. *Genome Biol* 2014, **15**(2):R29.
